# The effects of unsaturated fatty acids on psoriasis: A two‐sample Mendelian randomization study

**DOI:** 10.1002/fsn3.3543

**Published:** 2023-07-05

**Authors:** Hao Lei, Xin Chen, Baochen Cheng, Liumei Song, Ruiting Luo, Shengbang Wang, Tong Kang, Qian Wang, Yan Zheng

**Affiliations:** ^1^ Department of Dermatology The First Affiliated Hospital of Xi'an Jiaotong University Xi'an China; ^2^ State Key Laboratory of Military Stomatology & National Clinical Research Center for Oral Diseases & Shaanxi Clinical Research Center for Oral Diseases, Department of Orthodontics, School of Stomatology The Fourth Military Medical University Xi'an China; ^3^ Tangdu Hospital, Air Force Military Medical University Xi'an China

**Keywords:** causality, Mendelian randomization, psoriasis, single nucleotide polymorphisms, unsaturated fatty acids

## Abstract

Unsaturated fatty acids have been reported to be associated with the risk of psoriasis. However, the causal relationship between them remains unclear This study aimed to explore the causal relationship between unsaturated FAs and psoriasis. Firstly, we obtained genome‐wide association study (GWAS) data for psoriasis from the FINNGEN database (number of cases = 4510, number of controls = 212,242) and different FA levels (number of samples = 114,999) from the IEU OpenGWAS Project. Secondly, the genetic correlation coefficient was calculated using linkage disequilibrium fractional regression. Thirdly, a two‐sample Mendelian randomization (MR) analysis was performed using independent instrumental variables (*p* < 5 × 10^−8^) to determine the direction of randomization. Finally, expression quantitative trait loci (eQTL)‐related analyses of common single nucleotide polymorphisms (SNPs) were carried out to explore the potential molecular mechanisms of unsaturated FAs affecting psoriasis. We found that an increase in the ratio of monounsaturated fatty acids (MUFAs) to total fatty acids could increase the risk of psoriasis (inverse‐variance weighted [IVW], adjusted odds ratio [OR] = 1.175; adjusted 95% confidence interval [CI] = 1.045–1.321; adjusted *p* = .007). However, an increase in the ratio of polyunsaturated fatty acids (PUFAa) to total fatty acids could decrease the risk of psoriasis (IVW, adjusted OR = 0.754; adjusted 95% CI = 0.631–0.901; adjusted *p* = .002). Moreover, an increase in the ratio of PUFAs to MUFAs could decrease the risk of psoriasis (IVW, adjusted OR = 0.823; adjusted 95% CI = 0.715–0.948; adjusted *p* = .007). The heterogeneity of data was eliminated, and pleiotropy was not detected. There was no statistical difference in the MR analysis of other fatty acids indices with psoriasis. Further, no statistically significant evidence was found to verify a causal relationship between psoriasis and fatty acid levels in reverse MR. Functional enrichment analysis showed that these eQTL related to common SNPs were mainly involved in organic ion transport, choline metabolism, and the expression of key metabolic factors mediated by PKA, ChREBP, and PP2A. Our study indicated that the ratio of MUFAs to total fatty acids had a positive causal effect on psoriasis, while the ratio of PUFAs to total fatty acids and the ratio of PUFAs to MUFAs had a negative causal effect on psoriasis. Moreover, PKA‐, PP2A‐, and ChREBP‐mediated activation of metabolic factors may play an important role in this process.

## Graphical abstract

Our study indicated the ratio of monounsaturated fatty acids to total had a positive causal effect on psoriasis, while the ratio of PUFAs to total fatty acids and the ratio of PUFAs to monounsaturated fatty acids had a negative causal effect on psoriasis.

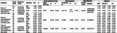

## 1 INTRODUCTION

Psoriasis, affecting roughly 2% of European and North American populations, is an immune‐mediated inflammatory skin disease (Boehncke & Schön, 2015; Paller et al., 2018). It is characterized by abnormal and rapid keratinocyte differentiation and thickened epidermis (Furue et al., 2018). There are many risk factors, such as genetics, infections, trauma, smoking, alcohol abuse, and stress, contributing to the development of psoriasis (Armstrong, 2014; Rendon & Schäkel, 2019; Tsoi et al., 2017). Various systemic diseases are strongly associated with it, including obesity, hypertension, hyperlipidemia, diabetes, metabolic syndrome, and cardiovascular diseases, which increase the disease burden (Armstrong et al., 2012, 2013; Boehncke et al., 2011). Further, a study showed that people with psoriasis were 1.5 times more likely to experience depressive symptoms and had a higher (20%–50%) incidence of anxiety symptoms (Hedemann et al., 2022). Based on the double burden of psoriasis on a patient's body and mind, the prevention and treatment of the disease are crucial.

Fatty acids (FAs), analyzed from their chemical structures, belong to a class of carboxylic acids, which includes saturated FAs (SFAs) and unsaturated FAs (Ratnayake & Galli, 2009). Among them, FAs containing double bonds in the chain are called unsaturated FAs, which are considered to have higher nutritional value (Lee & Park, 2014). Unsaturated FAs are divided into monounsaturated FAs (MUFAs) and polyunsaturated FAs (PUFAs) according to the number of double bonds they contain (Abedi & Sahari, 2014; Joffre et al., 2019; Ratnayake & Galli, 2009). Due to the existence of double bonds, they have stronger chemical reactivity; therefore, they have been widely studied. MUFAs contain only one double bond and have been reported to be associated with obesity, type II diabetes, and skin diseases (Imamura et al., 2016; Jiang et al., 2019; Sheashea et al., 2021). MUFAs play a dual role in anti‐inflammatory and pro‐inflammatory activities in the body (Khan & Jackson, 2018; Mika et al., 2018; J. Yang et al., 2020). PUFAs contain two or more double bonds, which are involved in the formation of cell membranes and regulate their fluidity and functions of cell membranes. Studies have reported that PUFAs are closely related to cancer, diabetes, cardiovascular diseases, nervous system diseases, and anti‐inflammatory responses (Brown et al., 2019; D'Eliseo & Velotti, 2016; Fischer et al., 2014; Joffre et al., 2019; Marklund et al., 2019; Zhang et al., 2020).

In dermatology, many studies have examined the relationship between skin diseases and FA levels. A study showed that elevated omega‐3 FA levels, a type of PUFAs, may increase the risk of systemic lupus erythematosus (Wang et al., 2022). Another study examined the serum FAs profile in psoriasis and its comorbidity to find the association of different FAs with psoriasis severity and comorbidity (Mysliwiec et al., 2017). Moreover, N‐3 polyunsaturated fatty acids (PUFAs) can promote wound healing after sunburn in mice (Meng et al., 2021). Although the meaningful roles of MUFAs and PUFAs in psoriasis have been discussed, the causal associations of these FAs with psoriasis remain unclear.

Mendelian randomization (MR) analysis is an emerging epidemiological technique that can take genetic variations as instrumental variables (IVs) to explore causal relationships between exposure factors and health outcomes. Compared with traditional observational studies, MR studies are less prone to confounding and reverse causation (Bennett & Holmes, 2017; Evans & Davey Smith, 2015). Among these studies, the two‐sample MR (TSMR) analysis allows researchers to extract genetic effect estimates from two nonoverlapping populations, which will effectively improve the reliability of causal inference.

Therefore, in this study, we performed a TSMR analysis on two independent genome‐wide association study (GWAS) datasets to explore the causal relationship between different FA levels and psoriasis.

## 2 RESULTS

### 2.1 Selection of instrumental variables

The details of strongly correlated IVs are presented in our Supplementary Tables, including the ratio of MUFAs to total FAs with 72 single nucleotide polymorphisms (SNPs) (Table S1), and the ratio of PUFAs to total FAs with 51 SNPs (Table S2), the ratio of PUFAs to MUFAs with 62 SNPs (Table S3). In the reverse MR analysis, we observed 16 SNPs for psoriasis (Table S4). The genetic strength was sufficient with all F‐value>10, except rs2021511 for psoriasis.

### 2.2 Monounsaturated fatty acids (MUFAs) and psoriasis

**FIGURE 1:**
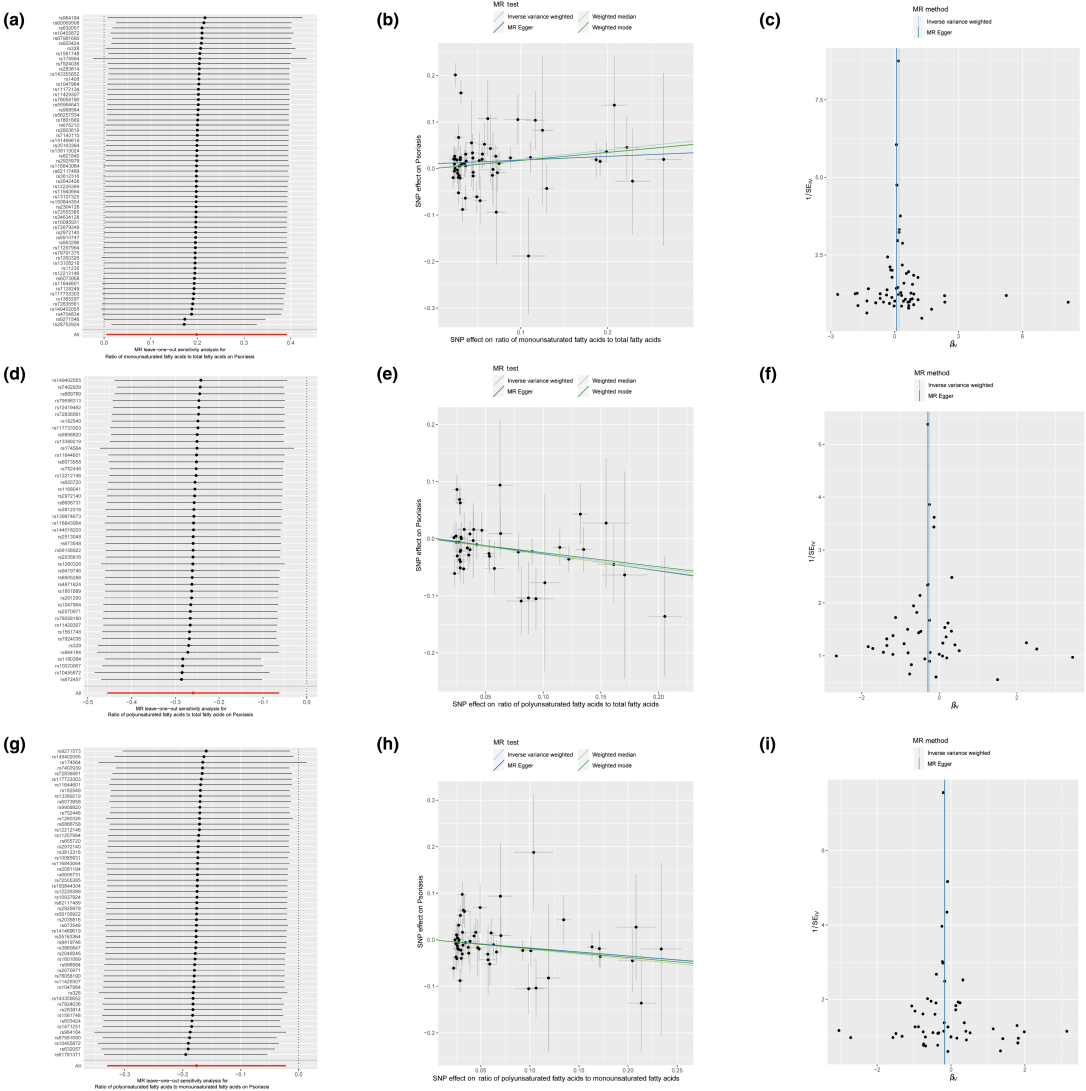
Mendelian randomization (MR) analysis of the causality of the ratio of unsaturated fatty acids with the risk of psoriasis. Sensitivity analysis (a), scatter plot (b), and funnel plot (c) for the causality of the ratio of monounsaturated fatty acids to total fatty acids with the risk of psoriasis. Sensitivity analysis (d), scatter plot (e), and funnel plot (f) for the causality of the ratio of polyunsaturated fatty acids to total fatty acids with the risk of psoriasis. Sensitivity analysis (g), scatter plot (h), and funnel plot (i) for the causality of the ratio of polyunsaturated fatty acids to monounsaturated fatty acids with the risk of psoriasis.

In our study, 66 SNPs were recorded in the MR preanalysis data from a combination of the ratio of MUFAs to total FAs regarding psoriasis (Table S5). In this process, five palindromic and incompatible SNPs were removed (rs1047964, rs1561748, rs28752924, rs28601761, and rs3789849). Hence, there were 61 SNPs that were involved in the MR analysis. The leave‐one‐out analysis suggested that the risk estimates of the ratio of MUFAs to total FAs regarding psoriasis generally remained consistent after eliminating each single SNP at a time (Figure 1a), which showed the sufficient sensitivity of our assay. The inverse‐variance‐weighted (IVW) test indicated that the increase in the ratio of MUFAs to total FAs might increase the risk of psoriasis (odds ratio [OR] =1.220; *p* = .044) (Table 2 and Figure 1b). A heterogeneity was detected between the individual SNP estimates using Cochran's *Q* test (*Q* = 165.114, *p* = 9.31 E‐12). After deleting the outliers detected using the MR‐PRESSO analysis, we reconducted the MR analysis (Figures S1–S3), which indicated the same trend as before (IVW, adjusted OR = 1.175, adjusted 95% confidence interval [CI] = 1.045–1.321; adjusted *p* = .007) and the heterogeneity was eliminated (Cochran's *Q* test p* = .510) (Table 2). The horizontal pleiotropy in MUFAs was not found with the MR‐Egger regression test (Egger intercept = 0.01, *p* = .292). Further, the symmetry of the funnel plot also indicated the same result (Figure 1c). The result of the weighted mode test (adjusted OR = 1.181; adjusted 95% CI = 1.016–1.374; adjusted *p* = .035) (Table 2) was consistent with that of the IVW test, which also indicated that the ratio of MUFAs to total FAs could increase the risk of psoriasis. In the reverse MR analysis regarding psoriasis and the ratio of MUFAs to total FAs, the IVW test showed no causality (OR = 1.004; 95% CI = 0.990–1.018; *p* = .578) (Table 3 and Figures S4–S6).

### 2.3 Polyunsaturated fatty acids and psoriasis

Forty‐eight SNPs were recorded in the MR preanalysis data from a combination of the ratio of PUFAs to total FAs regarding psoriasis (Table S6). In this process, three palindromic and incompatible SNPs were removed (rs1047964, rs1561748, and rs28601761). Hence, there were 45 SNPs were involved in the MR analysis. The leave‐one‐out analysis suggested that the risk estimates of the ratio of PUFAs to total FAs regarding psoriasis generally remained consistent after eliminating each single SNP at a time (Figure 1d). The IVW test indicated that the increase in the ratio of PUFAs to total FAs might decrease the risk of psoriasis (OR = 0.772; *p* = .010) (Table 2 and Figure 1e). A heterogeneity was detected between the individual SNP estimates using Cochran's *Q* test (*Q* = 65.296; *p* = .020). After deleting the outliers using the MR‐PRESSO analysis, we reconducted the MR analysis (Figures S7–S9), which showed the same trend as before (IVW, adjusted OR = 0.754; adjusted 95% CI = 0.631–0.901; adjusted *p* = .002) and the heterogeneity was eliminated (Cochran's *Q* test p* = .156) (Table 2). The horizontal pleiotropy in PUFAs was not observed with the MR‐Egger regression test (Egger intercept = 0.002; *p* = .818). The symmetry of the funnel plot also indicated the same result (Figure 1f). Further, the results of the weighted median test (Adjusted OR = 0.756; adjusted 95% CI = 0.605–0.945; adjusted *p* = .014) and weighted mode test (Adjusted OR = 0.778; adjusted 95% CI = 0.617–0.980; adjusted *p* = .039) (Table 2) were consistent with those of the IVW test. In the reverse MR analysis regarding psoriasis and the ratio of PUFAs to total FAs, the IVW test showed no causality (OR = 0.996; 95% CI = 0.984–1.008; *p* = .492) (Table 3 and Figures S10–S12).

### 2.4 Ratio of PUFAs to MUFAs and psoriasis

Here, 58 SNPs were recorded in the MR preanalysis data from a combination of the ratio of PUFAs to MUFAs and psoriasis (Table S7). In this process, three palindromic and incompatible SNPs were removed (rs1047964, rs1561748, and rs28601761). Therefore, 55 SNPs were involved in the MR analysis. The leave‐one‐out analysis suggested that the risk estimates of the ratio of PUFAs to MUFAs regarding psoriasis generally remained consistent after eliminating each single SNP at a time (Figure 1g). The IVW test indicated that the increase in the ratio of PUFAs to MUFAs may decrease the risk of psoriasis (OR = 0.839, *p* = .025) (Table 2 and Figure 1h). A heterogeneity was detected between individual SNP estimates using Cochran's *Q* test (*Q* = 77.329; *p* = .020). After deleting the outliers detected using the MR‐PRESSO analysis, we reconducted the MR analysis again (Figures S13–S15), which indicated the same trend as before (IVW, adjusted OR = 0.823; adjusted 95% CI = 0.715–0.948; adjusted *p* = .007) and the heterogeneity was eliminated (Cochran's *Q* test p* = .164) (Table 2). The horizontal pleiotropy in the ratio of PUFAs to MUFAs was not observed with the MR‐Egger regression test (Egger intercept = −0.0004, *p* = .957). Further, the symmetry of the funnel plot also indicated the same result (Figure 1i). The results of the weighted median test (Adjusted OR = 0.814; adjusted 95% CI = 0.672–0.986; adjusted *p* = .035) (Table 2) were consistent with that of the IVW test. In the reverse MR analysis of psoriasis and the ratio of PUFAs to MUFAs, the IVW test shows no causality (OR = 0.996; 95% CI = 0.983–1.009; *p* = .544) (Table 3 and Figures S16–S18).

### 2.5 Other fatty acid indices and psoriasis

From our analysis, the three indices above are strongly related to the risk of psoriasis. However, other indices are not associated with the risk of psoriasis based on the IVW test (*p* > .05), including total FAs, SFAs levels, PUFAs levels, omega‐3 FAs levels, omega‐6 FAs levels, the ratio of SFAs to total FAs, the ratio of omega‐3 FAs to total FAs, the ratio of omega‐6 FAs to total FAs, the ratio of omega‐6 FAs to omega‐3 FAs, the ratio of docosahexaenoic acid to total FAs, and the ratio of linoleic acid to total FAs (Table S8). The reverse MR analysis regarding psoriasis and the other FA indices suggested that the effect of psoriasis does not increase these FA indices (Table S9).

### 2.6 Potential pleiotropy searched in PhenoScanner

In the three meaningful indices above, 161 SNPs were involved in the MR analysis. We searched these SNPs in the PhenoScanner database. We found five main traits that may create potential pleiotropy, such as smoking or cigarette use, drinking or alcohol intake, cardiovascular disease, body mass index (BMI), and diabetes (Table S10). Therefore, we deleted each potential SNPs in the corresponding data and reconducted the MR analysis again. The IVW test result was consistent with what we previously observed, which indicated the same causality (Table S11).

### 2.7 The eQTL‐Related analysis of common SNPs

**FIGURE 2:**
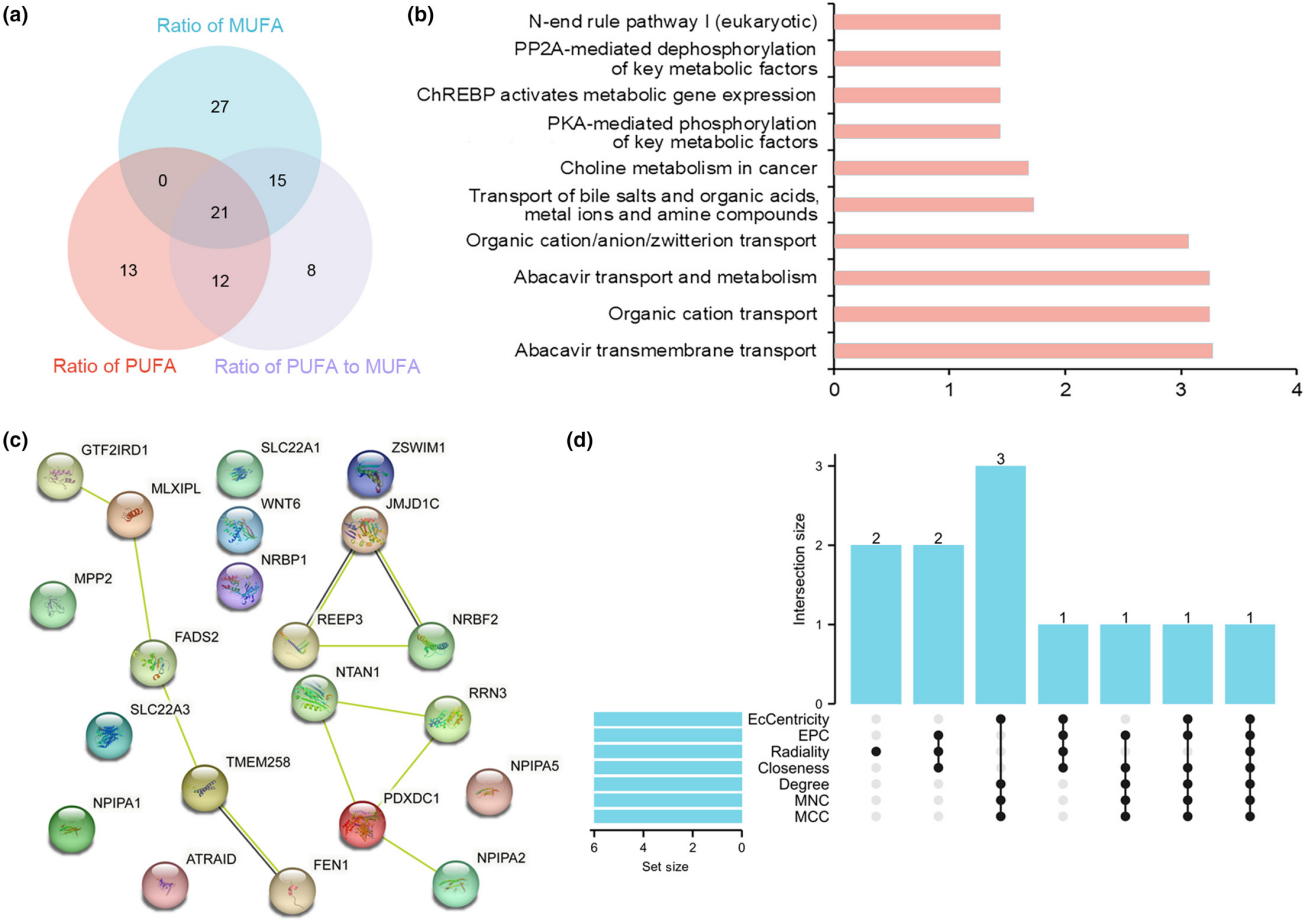
The expression quantitative trait loci (eQTL)‐Related Analysis of Common single nucleotide polymorphisms (SNPs). Venn diagram of common SNPs in three datasets (a); Functional enrichment analysis of eQTL (b); PPI network of eQTL (c); Upset diagram regarding the top six genes estimated by seven different algorithms (d).

We found 21 common SNPs through the intersection of the IVs of the three datasets (Table S12 and Figure 2a). Among them, 28 eQTL were closely related to 11 SNPs after removing duplicates (Table S13). The functional enrichment analysis showed that these eQTL were mainly involved in organic ion transport, choline metabolism, and the expression of key metabolic factors mediated by PKA, ChREBP, and PP2A (Table S14 and Figure 2b). Twelve eQTL had interactions among the 28 eQTL (Figure 2c). In the top six genes estimated by seven different algorithms, NRBF2 ranked highest in all the algorithms. Moreover, PDXDC1 and JMJD1C ranked highest in at least five algorithms (Table S15 and Figure 2d).

## 3 DISCUSSION

In our study, we found that the ratio of MUFAs to total FAs had a positive causal effect on psoriasis. In contrast, the ratio of PUFAs and the ratio of PUFAs to MUFAs had a negative causal effect on psoriasis. However, there seemed to be no causal relationship detected between the FAs and psoriasis, including total FAs, saturated FAs, polyunsaturated FAs, Omega‐3 FAs, Omega‐6 FAs, the ratio of saturated FAs to total FAs, the ratio of omega‐3 FAs to total FAs, the ratio of omega‐6 FAs to total FAs, the ratio of omega‐6 FAs to omega‐3 FAs, the ratio of docosahexaenoic acid to total FAs, and the ratio of linoleic acid to total FAs. Considering the limitations of the data volume and analytical methods, we focused on the three meaningful causal relationships in the following discussion.

Firstly, 61 IVs were obtained for the MR analysis regarding the ratio of MUFAs to total FAs and psoriasis. The IVW test and weighted mode tests indicated that the genetically elevated ratio of MUFAs to total FAs could increase the risk of psoriasis. No significant pleiotropy was found between the gene variant of the ratio of MUFAs to total FAs and psoriasis in the pleiotropic analysis, nor was there a single SNP that was statistically significant to the results detected in the leave‐one‐out analysis. Although statistical significance was observed in the MR‐Egger regression, weighted median, and simple mode tests, their ORs were >1, showing the same trend as the IVW and weighted mode tests. These results suggested that the gene variant of the ratio of MUFAs to total FAs may positively influence the pathogenesis of psoriasis through the ratio of MUFAs to total FAs itself rather than other pathways.

Secondly, 45 IVs were chosen for the MR analysis regarding the ratio of PUFAs to total FAs and psoriasis. Three tests showed that the genetically elevated ratio of PUFAs to total FAs could decrease the risk of psoriasis, except for the MR‐Egger regression and simple mode tests. Considering the results of the pleiotropy and leave‐one‐out test and the same trends of MR‐Egger regression and simple mode tests, we concluded that the gene variant of the ratio of PUFAs to total FAs might negatively influence the pathogenesis of psoriasis.

Thirdly, 55 IVs were involved in the MR analysis regarding the ratio of PUFAs to MUFAs and psoriasis. The IVW test and weighted median tests indicated that the genetically elevated ratio of PUFAs to MUFAs could decrease the risk of psoriasis. This result is consistent with the causal relationship between PUFAs and psoriasis, In other words, an increase in the ratio of PUFAs or a decrease in the ratio of MUFAs can lead to an increase in the ratio of PUFAs to MUFAs, which, in turn, negatively affects the risk of psoriasis. It also corroborates the reliability of our analysis.

Many studies have shown that the serum concentration and intake of unsaturated FAs are related to the onset of psoriasis. A serum FA profile analysis of 85 patients with active plaque psoriasis showed that PASI was associated with low circulating levels of DHA, n‐3 PUFAs, and high proportions of MUFAs. These findings suggest an abnormal FA profile in patients with psoriasis (Mysliwiec et al., 2017). Similar changes were found in the FA profiles of patients with psoriatic arthritis (Mysliwiec et al., 2019). A retrospective study of 82 clinical samples showed that the level of dietary MUFAs was the main predictor of the clinical severity of psoriasis (Barrea et al., 2015). Forty‐four patients with mild‐to‐severe plaque psoriasis receiving immunosuppressive drugs were randomly assigned to either a regular diet or an n3‐PUFAs‐enriched diet for 6 months and found that the latter showed greater clinical improvement (Guida et al., 2014). A prospective study showed that vitamin D supplementation for 5 years, with or without n‐3 FAs, was associated with a 22% reduction in autoimmune diseases, including psoriasis, rheumatoid arthritis, and so on (Hahn et al., 2022). A meta‐analysis of 13 randomized controlled trials (RCTs) in 2019 showed that fish oil supplementation, rich in n‐3 PUFAs, did not significantly reduce the severity of psoriasis (Yang & Chi, 2019). Further, a meta‐analysis of 18 RCTs involving 927 study participants in 2020 found that fish oil or omega‐3 PUFAs monotherapy had little effect on psoriasis area and severity indices; however, they could show significant adjunctive effects when combined with traditional treatments (Chen et al., 2020). A review suggested that eating fish rich in n‐3 PUFAs, as well as fruits and vegetables, are beneficial for people with psoriasis (Musumeci et al., 2022). Some researchers have pointed out that the roles of n‐3 and n‐6 PUFAs in most common skin diseases are opposite and uncertain, but they are not simply black and white. A combination of omega‐6 FAs, gamma‐linolenic acid (GLA), and omega‐3 long‐chain PUFAs supplements appears to have the highest potential for reducing inflammation (Balić et al., 2020). The above studies all point to the positive effects of MUFAs and the negative effects of PUFAs on psoriasis.

There are several potential mechanisms that could explain the causal link between unsaturated FAs and psoriasis. A transcriptome sequencing analysis of five patients with psoriasis showed that the PPAR FA metabolism pathway was significantly downregulated in patients with psoriasis, which could affect the inhibitory effect of PPAR on various inflammatory cytokines and transcription factors, such as AP‐1 and NF‐kB (Yu et al., 2020). As demonstrated in an in vitro model of psoriasis, DHA supplementation rebalances PPAR receptor expression and results in decreased TNF‐α secretion, thereby restoring the differentiation and proliferation of epidermal cells and reducing inflammation (Morin et al., 2021). It was proved in a mouse model of psoriasis that olive oil, belonging to PUFAs, can exacerbate psoriatic skin symptoms by inducing Nrf2 overexpression and an imbalance in oleic and linoleic acid levels (Donato‐Trancoso et al., 2022). Some researchers adopted fat‐1, a transgenic mouse that can endogenously convert n‐6 PUFAs to n‐3 PUFAs, to investigate the mechanism of action of n3 PUFAs in psoriasis. They found that n‐3 FAs stimulated Th17 cells to produce lower levels of inflammatory factors, including IL‐17, IL‐22, and IL‐23, and stimulated Treg cells to produce higher levels of anti‐inflammatory factors, such as Foxp3 (Qin et al., 2014). Similar to previous studies, we found that these eQTL are associated with PKA, PP2A, and ChREBP‐mediated activation of metabolic factors. Studies have shown that cold stimulation can initiate a signaling cascade of triglyceride hydrolysis and PKA activation, which interacts with nuclear receptors, such as PPAR, to enhance the breakdown capacity of FAs (Liu et al., 2019). In diabetic mice, unrestricted activation of PKA impairs the antioxidant defense system of mTORC1‐p62‐Keap1‐Nrf2, leading to increased oxidative stress (Liu et al., 2021). Activation of PPAR may increase the expression of PP2A (Muzio et al., 2007). Moreover, PP2A can upregulate the expression of Nrf2 and promote autophagy and apoptosis in cardiomyocytes (Guan et al., 2019). ChREBP is located in the promoter region of PPARγ, and its activation can induce the activation of genes related to adipogenesis and autophagy (Zhao et al., 2020). Furthermore, we found that the key gene NRBF2 acts as a ligand for PPAR, activating the expression of PPAR (Yasumo et al., 2000).

This study had several strengths. Firstly, the study design was based on three main assumptions of IVs and conformed to the MR analysis process, which guaranteed the reliability of the conclusion. Secondly, both large GWAS data were obtained from European populations and were independent of each other, which avoided bias, including sample size, population, and ethnicity. Thirdly, we employed a total of five main MR analysis methods to evaluate the consistency of causality.

However, this study had some limitations. First, the populations of the two GWAS databases were all Europeans; therefore, the extrapolation of the results was limited, and the corresponding GWAS datasets are needed for populations in Asia, the Americas, etc. Second, the GWAS data were derived from public databases. The details of patients, including age, sex, and disease severity were unknown, so only a relatively macroscopic analysis can be performed. Third, in the selection of IVs, eliminating linkage disequilibrium (LD) and detecting pleiotropy can only reduce the influence of internal factors, such as biological mechanisms and genetic co‐inheritance, but cannot eliminate them. Finally, this study only used two classic large‐sample databases, and there may have been omissions in data retrieval, which may have affected the reliability of the analysis results.

The n‐3 and n‐6 PUFAs may play different roles in psoriasis; however, the change of n‐6 PUFAs in serum FA profile of patients with psoriasis is not significant. Therefore, the ratio of PUFAs to total FAs in serum is negatively related to that of psoriasis from this perspective. The positive causal relationship between the ratio of MUFAs to total FAs and psoriasis is consistent with our study.

## 4 METHODS

### 4.1 Data source

In this study, the genetic variables for psoriasis (case = 4510, control = 212,242) were obtained from the FINNGEN database (https://www.finngen.fi/en). The genetic variables for different kinds of FA levels (*N* = 114,999) were obtained from data regarding the IEU OpenGWAS Project (https://gwas.mrcieu.ac.uk/) including the level of total FAs, saturated FAs, polyunsaturated FAs, omega‐3 FAs, omega‐6 FAs, and the ratio between specific FAs. The population from which the data regarding psoriasis and FA levels were obtained were European, belonging to different cohorts. The details regarding the data source are presented in Table 1. Choosing two different databases ensured that the study population did not overlap.

**TABLE 1:** Summary of genome‐wide association study (GWAS) datasets in our study.

Trait	GWAS ID	Database	Population	Sample size	Number of SNPs
Psoriasis	finn‐b‐L12_PSORIASIS	FINNGEN	European	216,752	16,380,464
Total fatty acids	met‐d‐Total_FA	IEU open GWAS project	European	114,999	12,321,875
Saturated fatty acids	met‐d‐SFA		European	114,999	12,321,875
Polyunsaturated fatty acids	met‐d‐PUFA		European	114,999	12,321,875
Omega‐3 fatty acids	met‐d‐Omega_3		European	114,999	12,321,875
Omega‐6 fatty acids	met‐d‐Omega_6		European	114,999	12,321,875
Ratio of saturated fatty acids to total fatty acids	met‐d‐SFA_pct		European	114,999	12,321,875
Ratio of monounsaturated fatty acids to total fatty acids	met‐d‐MUFA_pct		European	114,999	12,321,875
Ratio of polyunsaturated fatty acids to total fatty acids	met‐d‐PUFA_pct		European	114,999	12,321,875
Ratio of polyunsaturated fatty acids to monounsaturated fatty acids	met‐d‐PUFA_by_MUFA		European	114,999	12,321,875
Ratio of omega‐3 fatty acids to total fatty acids	met‐d‐Omega_3_pct		European	114,999	12,321,875
Ratio of omega‐6 fatty acids to total fatty acids	met‐d‐Omega_6_pct		European	114,999	12,321,875
Ratio of omega‐6 fatty acids to omega‐3 fatty acids	met‐d‐Omega_6_by_Omega_3		European	114,999	12,321,875
Ratio of docosahexaenoic acid to total fatty acids	met‐d‐DHA_pct		European	114,999	12,321,875
Ratio of linoleic acid to total fatty acids	met‐d‐LA_pct		European	114,999	12,321,875

**TABLE 2:** MR analysis for the causality of unsaturated fatty acids with the risk of psoriasis.

Exposure	Total SNPs	Removed SNPs	Methods	OR	*p*	Horizontal pleiotropy			Heterogeneity			MR‐PRESSO		Adjusted			
						Egger intercept	se	*p*	Cochran's Q	*p*	*p* *	Global test *p*	Outliers	OR*	95% CI*		*p* *
Ratio of monounsaturated fatty acids to total fatty acids	66	rs1047964 rs1561748 rs28752924 rs28601761 rs3789849	ME	1.081	.605								rs28752924 rs60069508 rs9271546	1.169	0.978	1.398	.092
	66		WMd	1.191	.044									1.191	0.995	1.426	.057
	66		IVW	1.220	.044	0.010	0.010	.292	165.114	9.31 E‐12	.510	<2e‐04		1.175	1.045	1.321	.007
	66		SM	1.279	.103									1.279	0.948	1.726	.113
	66		Wmo	1.189	.028									1.181	1.016	1.374	.035
Ratio of polyunsaturated fatty acids to total fatty acids	48	rs1047964 rs1561748 rs28601761	ME	0.746	.107								rs1180384	0.790	0.575	1.085	.152
	48		WMd	0.758	.025									0.756	0.605	0.945	.014
	48		IVW	0.772	.010	0.002	0.010	.818	65.296	.020	.156	0.029		0.754	0.631	0.901	.002
	48		SM	0.807	.312									0.799	0.552	1.157	.242
	48		Wmo	0.782	.043									0.778	0.617	0.980	.039
Ratio of polyunsaturated fatty acids to monounsaturated fatty acids	58	rs1047964 rs1561748 rs28601761	ME	0.843	.181								rs61781371	0.865	0.691	1.083	.212
	58		WMd	0.814	.039									0.814	0.672	0.986	.035
	58		IVW	0.839	.025	−0.0004	0.008	.957	77.329	.020	.164	0.030		0.823	0.715	0.948	.007
	58		SM	0.790	.106									0.793	0.579	1.088	.157
	58		Wmo	0.827	.030									0.836	0.701	0.997	.051

*Note*: Removed SNPs include palindromic and incompatible allele SNPs.

Abbreviations: IVW, Inverse‐variance weighted; ME, MR‐Egger; SM, Simple mode; WMd, Weighted median; Wmo, Weighted mode.

* Represents adjusted value.

**TABLE 3:** MR analysis for the causality of psoriasis with the risk of unsaturated fatty acids.

Outcome	Total SNPs	Removed SNPs	Methods	OR	95% CI		SE	*p*	Horizontal pleiotropy			Heterogeneity	
									Egger_intercept	se	*p*	Cochran's Q	*p*
Ratio of monounsaturated fatty acids to total fatty acids	14	rs28752856 rs2021511	ME	1.013	0.992	1.034	0.011	.255					
	14		WMd	1.013	1.000	1.026	0.006	.043					
	14		IVW	1.004	0.990	1.018	0.007	.578	−0.004	0.003	.295	20.258	.042
	14		SM	1.006	0.977	1.036	0.015	.708					
	14		Wmo	1.014	1.002	1.027	0.006	.049					
Ratio of polyunsaturated fatty acids to total fatty acids	14	rs28752856 rs2021511	ME	0.991	0.972	1.011	0.010	.410					
	14		WMd	0.990	0.977	1.002	0.007	.112					
	14		IVW	0.996	0.984	1.008	0.006	.492	0.002	0.003	.586	16.126	.137
	14		SM	0.993	0.965	1.023	0.015	.662					
	14		Wmo	0.988	0.974	1.002	0.007	.121					
Ratio of polyunsaturated fatty acids to monounsaturated fatty acids	14	rs28752856 rs2021511	ME	0.989	0.968	1.009	0.011	.305					
	14		WMd	0.986	0.973	0.998	0.006	.023					
	14		IVW	0.996	0.983	1.009	0.007	.544	0.003	0.003	.385	19.150	.058
	14		SM	0.993	0.967	1.021	0.014	.634					
	14		Wmo	0.986	0.974	0.999	0.007	.058					

*Note*: rs28752856 is removed because of incompatible alleles; rs2021511 is removed because of incompatible allele negative *F* value.

Abbreviations: IVW, Inverse‐variance weighted; ME, MR‐Egger; SM, Simple mode; WMd, Weighted median; Wmo, Weighted mode.

### 4.2 Selection of instrumental variables

In this study, the criteria for screening the IVs from the genetic variation were as follows: (1) SNPs were closely associated with serum FAs levels; (2) SNPs were not associated with confounders of psoriasis and serum FAs levels; (3) SNPs can only affect psoriasis through FAs levels, and there is no direct connection between SNPs and psoriasis (Gagliano Taliun & Evans, 2021). The screening steps for IVs strongly associated with serum FAs levels are as follows: Firstly, we extracted SNPs associated with FAs levels by *p* < 5 × 10–8. To ensure the independence of instrumental variables, we excluded SNPs that were in linkage disequilibrium (LD) (*r* ^2^ < 0.001; clumping window =10,000 kb). Then, we extracted the above IVs in the SNPs of psoriasis and reconciled the exposure data and the outcome data to ensure that the effect of the SNPs on the exposure and the outcome corresponded to the same alleles. Palindromic and incompatible allele SNPs were excluded. We also calculated the F‐statistic to eliminate the bias caused by weak IVs in the results. The F‐statistic was calculated as *F* = *R* ^2^ (n‐k‐1)/[k (1‐*R* ^2^)]. The *R* ^2^ statistic was calculated using the R package “get_r_from_pn”, which reflects the degree to which the instrumental variable explains the exposure. In the reverse MR analysis, the *R* ^2^ statistic was calculated using the R package “get_r_from_lor”.

### 4.3 MR analysis

The R package “TwoSampleMR” was used to analyze the causality of unsaturated FAs with the risk of psoriasis. Five different regression models were chosen to identify the causation, including IVW, MR‐Egger, weighted median, simple mode, and weighted mode. Among them, the IVW test was the main method because of its stability. In the IVW test, we considered that the IVs were not pleiotropic, and the results of the GWAS were mostly phenotype standardized. Moreover, we considered that there was a proportional relationship between outcome and exposure. Therefore, we ensured that these SNPs were not pleiotropic when using the IVW test; otherwise, the results would have been greatly biased (Burgess et al., 2013). Compared with the IVW test, the MR‐Egger method considers the existence of the intercept term, which can be used as a supplement to the IVW test, but may be biased and exaggerate the type I error (Burgess & Thompson, 2017). Further, we used the weighted median, simple mode, and weighted mode tests to accumulate more evidence. We mainly focus on the analysis results of the IVW test in the MR analysis and further adopted methods such as sensitivity and heterogeneity analysis to enhance the reliability of the results.

### 4.4 Sensitivity analysis

Firstly, the MR‐Egger regression test was used to detect pleiotropy. If the *p*‐value was >.05, there was no pleiotropy in the data even if the intercept term was not 0. Then, Cochran's *Q* test was used to test for heterogeneity between two sets of data. Further, the Mendelian Randomization Pleiotropy Residual Sum and Outlier (MR‐PRESSO) test was used to detect and correct for horizontal pleiotropy. After deleting the outliers of IVs, we reconducted MR analysis with heterogeneity and pleiotropy tests. Finally, we used the leave‐one‐out method for sensitivity analysis to further verify the stability of our results. What is more, we retrieved the potential secondary phenotypes of each IV from PhenoScanner (http://www.phenoscanner.medschl.cam.ac.uk/), excluded IVs that might control confounding traits and reconducted MR analysis.

### 4.5 The eQTL‐Related analysis of common SNPs

To further explore the potential molecular mechanisms of unsaturated FAs affecting psoriasis, we firstly used Venn diagrams to identify common SNPs in three datasets, including met‐d‐MUFA_pct, met‐d‐PUFA_pct, and met‐d‐PUFA_by_MUFA. Then, these common SNPs were searched in the QTLs database for eQTL individually (http://www.mulinlab.org/qtlbase/index.html), and the tissue type was limited to the skin (Zheng et al., 2020). Thirdly, we performed a functional enrichment analysis based on the KOBAS‐i database (http://bioinfo.org/kobas/) (Bu et al., 2021). Finally, the String database (https://cn.string‐db.org/) (Szklarczyk et al., 2023) was used to explore the interaction among these eQTL, and Cytoscape was used to identify the hub genes through seven different algorithms.

## 5 CONCLUSION

Our study indicated that the ratio of MUFAs to total FAs had a positive causal effect on psoriasis, while the ratio of PUFAs to total FAs and the ratio of PUFAs to MUFAs had a negative causal effect on psoriasis. PKA, PP2A, and ChREBP‐mediated activation of metabolic factors may play an important role in this process. However, there seemed to be no causal relationship detected between the other FAs and psoriasis, including total FAs, SFAs levels, polyunsaturated FAs levels, Omega‐3 FAs levels, Omega‐6 FAs levels, and the specific ratios mentioned in the results. Meanwhile, our study verified the association of unsaturated FAs with psoriasis from the perspective of genetic variation, hoping to provide data support for the study of the role of FAs in psoriasis.

## AUTHOR CONTRIBUTIONS

**Hao Lei:** Conceptualization (equal); formal analysis (equal). **Xin Chen:** Formal analysis (equal). **Baochen Cheng:** Writing – original draft (equal). **Liumei Song:** Writing – original draft (equal). **Ruiting Luo:** Visualization (equal). **Shengbang Wang:** Visualization (equal). **Tong Kang:** Writing – review and editing (equal). **Qian Wang:** Writing – review and editing (equal). **Yan Zheng:** Conceptualization (lead).

## Supporting information


Figures S1–S18.

Tables S1–S15.

## Data Availability

The data that support the findings of this study are available in the public database mentioned in this manuscript.
