# Supplementary material for: The effects of unsaturated fatty acids on psoriasis: A two‐sample Mendelian randomization study
Source: Food Sci Nutr. 2023 Jul 5;11(10):6073–84. doi: 10.1002/fsn3.3543 (PMC10563715; doi:10.1002/fsn3.3543)
Supplement: Supplementary file 1 — Figures S1–S18. [file FSN3-11-6073-s001.pdf]

Supplementary Figures

Fig S1: Sensitivity analysis for the causality of Ratio of monounsaturated acids to total fatty acids with the risk of Psoriasis after deleted the pleiotropic snps

Fig S2: Scatter plot for the causality of Ratio of monounsaturated acids to total fatty acids with the risk of Psoriasis after deleted the pleiotropic snps

Fig S3: Funnel plot for the causality of Ratio of monounsaturated acids to total fatty acids with the risk of Psoriasis after deleted the pleiotropic snps

Fig S4: Sensitivity analysis for the causality of Psoriasis with the Ratio of monounsaturated fatty acids to total fatty acids

Fig S5: Scatter plot for the causality of Psoriasis with the Ratio of monounsaturated fatty acids to total fatty acids

Fig S6: Funnel plot for the causality of Psoriasis with the Ratio of monounsaturated fatty acids to total fatty acids

Fig S7: Sensitivity analysis for the causality of Ratio of polyunsaturated acids to total fatty acids with the risk of Psoriasis after deleted the pleiotropic snps

Fig S8: Scatter plot for the causality of Ratio of polyunsaturated acids to total fatty acids with the risk of Psoriasis after deleted the pleiotropic snps

Fig S9: Funnel plot for the causality of Ratio of polyunsaturated acids to total fatty acids with the risk of Psoriasis after deleted the pleiotropic snps

Fig S10: Sensitivity analysis for the causality of Psoriasis with the Ratio of polyunsaturated fatty acids to total fatty acids

Fig S11: Scatter plot for the causality of Psoriasis with the Ratio of polyunsaturated fatty acids to total fatty acids

Fig S12: Funnel plot for the causality of Psoriasis with the Ratio of polyunsaturated fatty acids to total fatty acids

Fig S13: Sensitivity analysis for the causality of Ratio of polyunsaturated fatty acids to monounsaturated fatty acids with the risk of Psoriasis after deleted the pleiotropic snps

Fig S14: Scatter plot for the causality of Ratio of polyunsaturated fatty acids to monounsaturated fatty acids with the risk of Psoriasis after deleted the pleiotropic snps

Fig S15: Funnel plot for the causality of Ratio of polyunsaturated fatty acids to monounsaturated fatty acids with the risk of Psoriasis after deleted the pleiotropic snps

Fig S16: Sensitivity analysis for the causality of Psoriasis with Ratio of polyunsaturated fatty acids to monounsaturated fatty acids

Fig S17: Scatter plot for the causality of Psoriasis with Ratio of polyunsaturated fatty acids to monounsaturated fatty acids

Fig S18: Funnel plot for the causality of Psoriasis with Ratio of polyunsaturated fatty acids to monounsaturated fatty acids

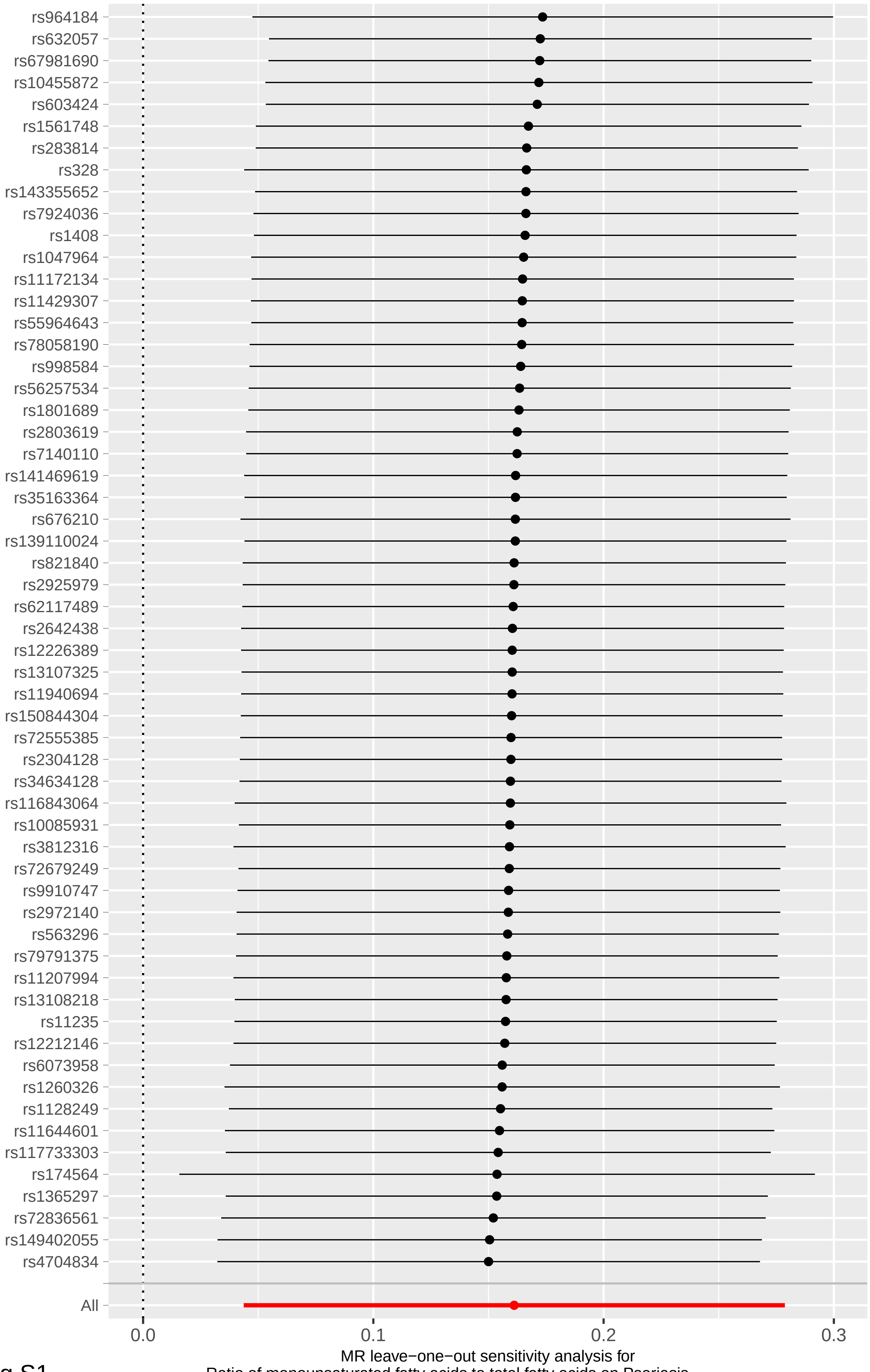
rs964184
rs632057
rs67981690
rs10455872
rs603424
rs1561748
rs283814
rs328
rs143355652
rs7924036
rs1408
rs1047964
rs11172134
rs11429307
rs55964643
rs78058190
rs998584
rs56257534
rs1801689
rs2803619
rs7140110
rs141469619
rs35163364
rs676210
rs139110024
rs821840
rs2925979
rs62117489
rs2642438
rs12226389
rs13107325
rs11940694
rs150844304
rs72555385
rs2304128
rs34634128
rs116843064
rs10085931
rs3812316
rs72679249
rs9910747
rs2972140
rs563296
rs79791375
rs11207994
rs13108218
rs11235
rs12212146
rs6073958
rs1260326
rs1128249
rs11644601
rs117733303
rs174564
rs1365297
rs72836561
rs149402055
rs4704834
All
0.0
0.1
0.2
0.3
MR leave−one−out sensitivity analysis for
Ratio of monounsaturated fatty acids to total fatty acids on Psoriasis

Fig S1

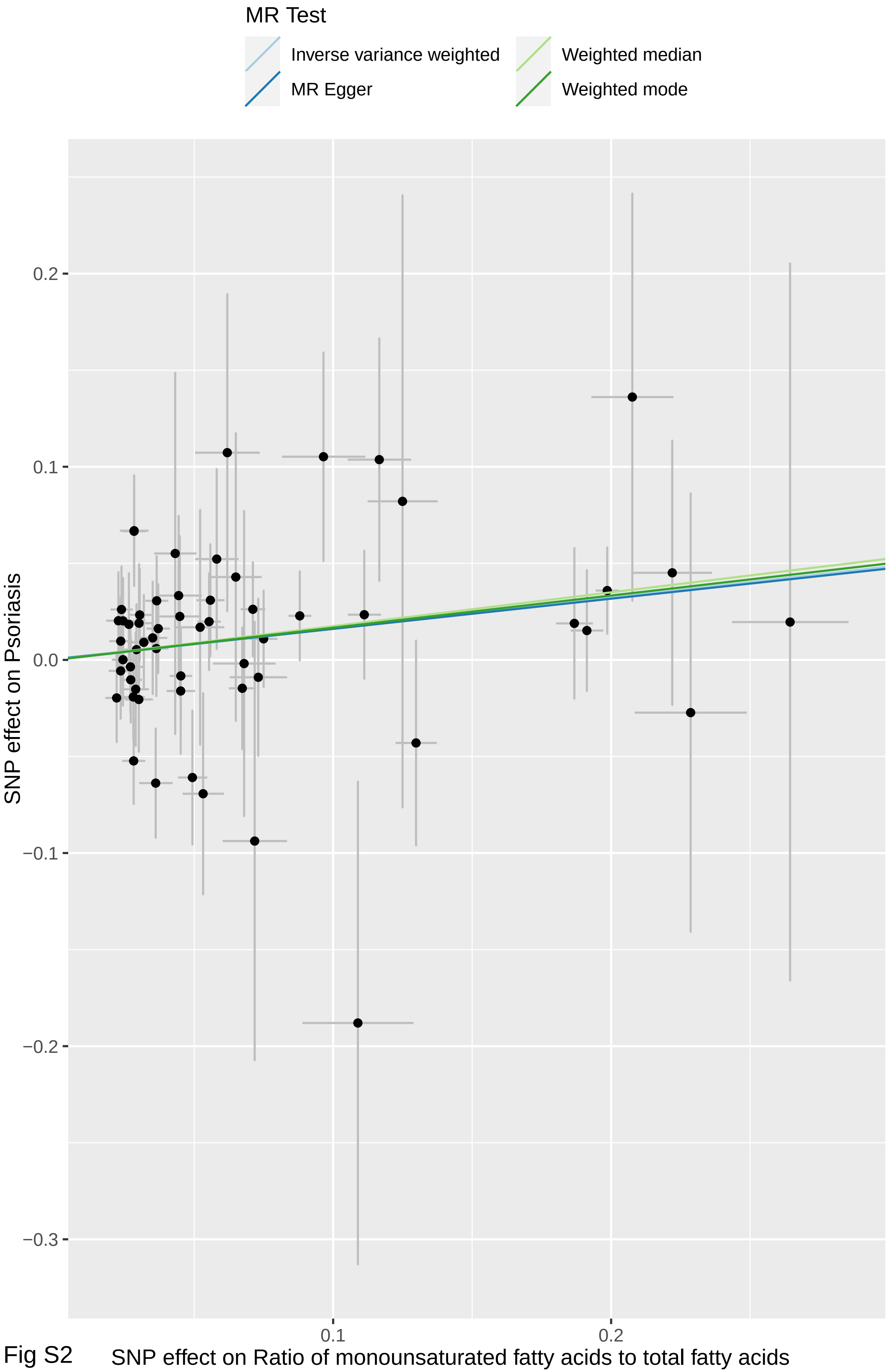
MR Test
Inverse variance weighted
MR Egger
Weighted median
Weighted mode
0.2
0.1
0.0
−0.1
−0.2
−0.3
SNP effect on Psoriasis
0.1
0.2
SNP effect on Ratio of monounsaturated fatty acids to total fatty acids

Fig S2

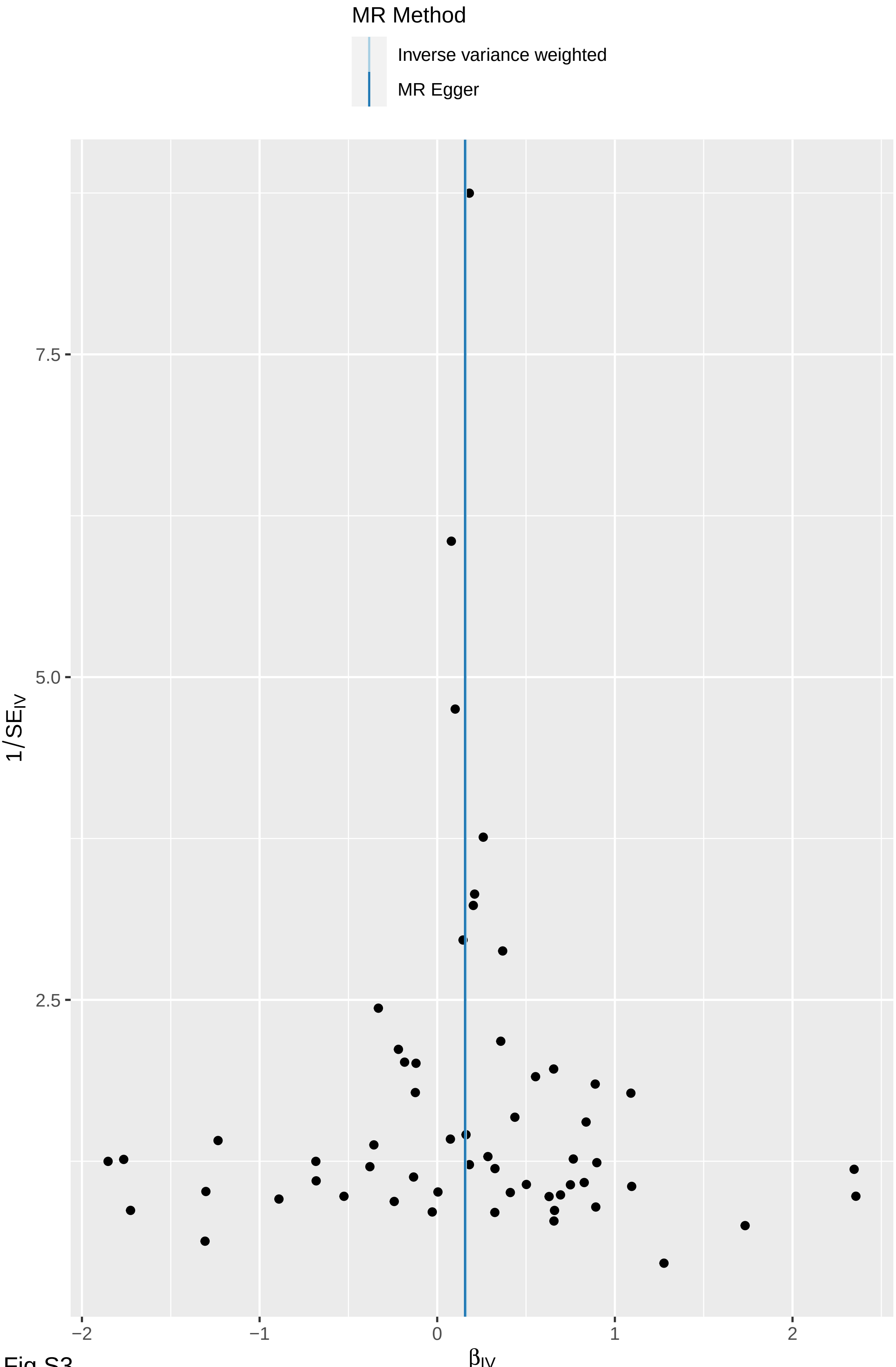
MR Method
Inverse variance weighted
MR Egger
7.5
5.0
2.5
1/SE_IV
−2
−1
0
1
2
β_IV

Fig S3

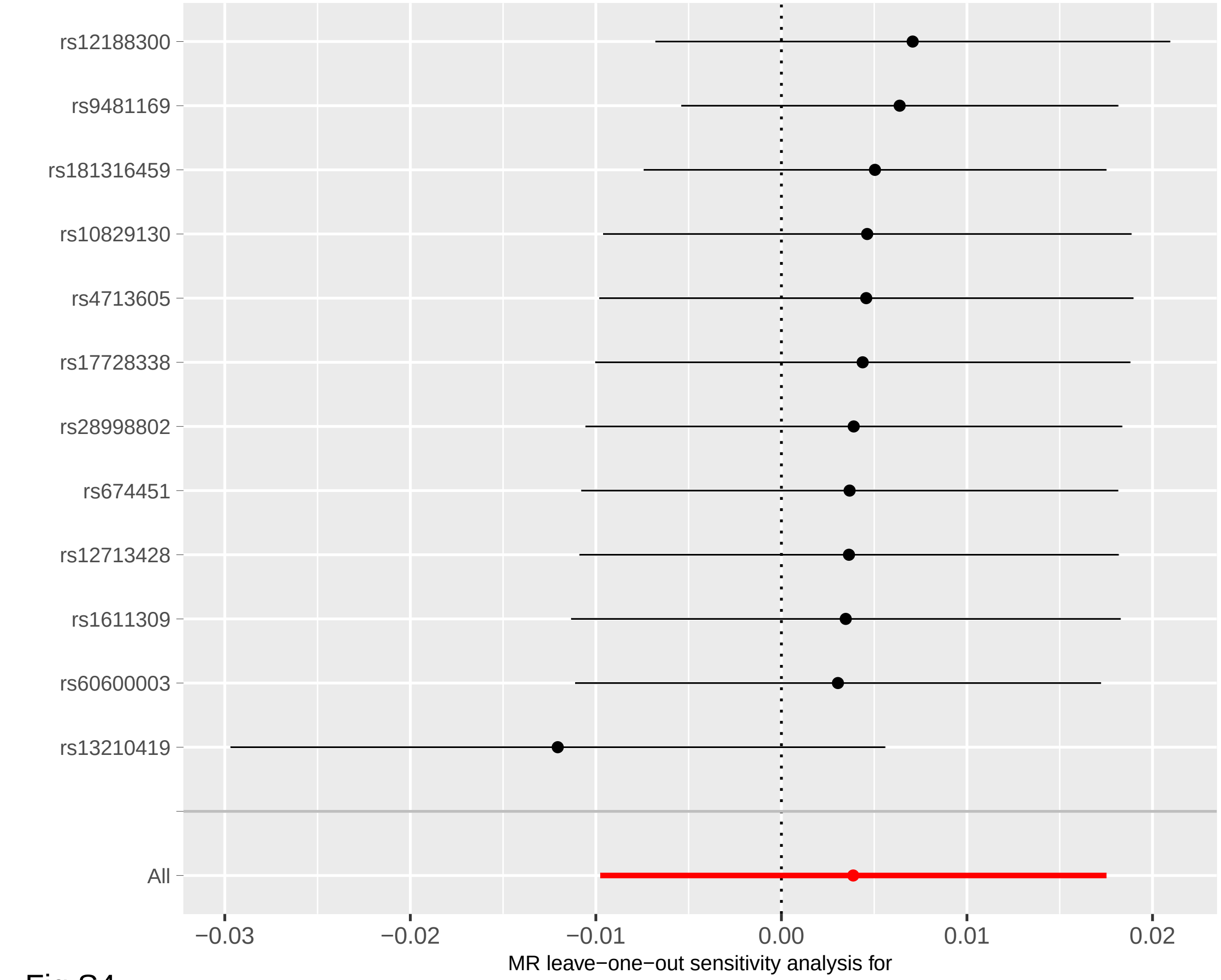
rs12188300
rs9481169
rs181316459
rs10829130
rs4713605
rs17728338
rs28998802
rs674451
rs12713428
rs1611309
rs60600003
rs13210419
All
−0.03
−0.02
−0.01
0.00
0.01
0.02
MR leave−one−out sensitivity analysis for
Psoriasis on Ratio of monounsaturated fatty acids to total fatty acids

Fig S4

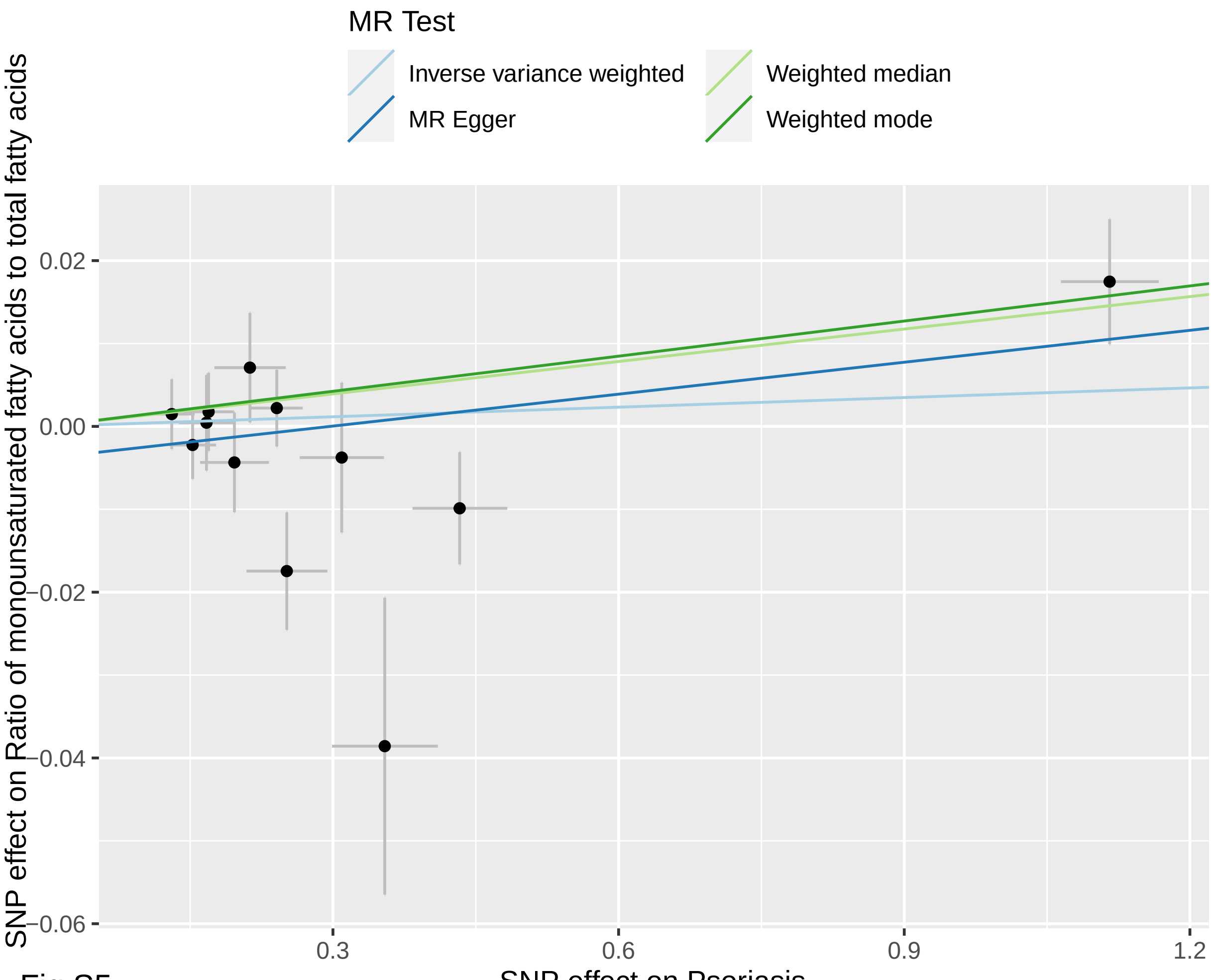
MR Test
Inverse variance weighted
Weighted median
MR Egger
Weighted mode
SNP effect on Ratio of monounsaturated fatty acids to total fatty acids
0.02
0.00
−0.02
−0.04
−0.06
0.3
0.6
0.9
1.2
SNP effect on Psoriasis

Fig S5

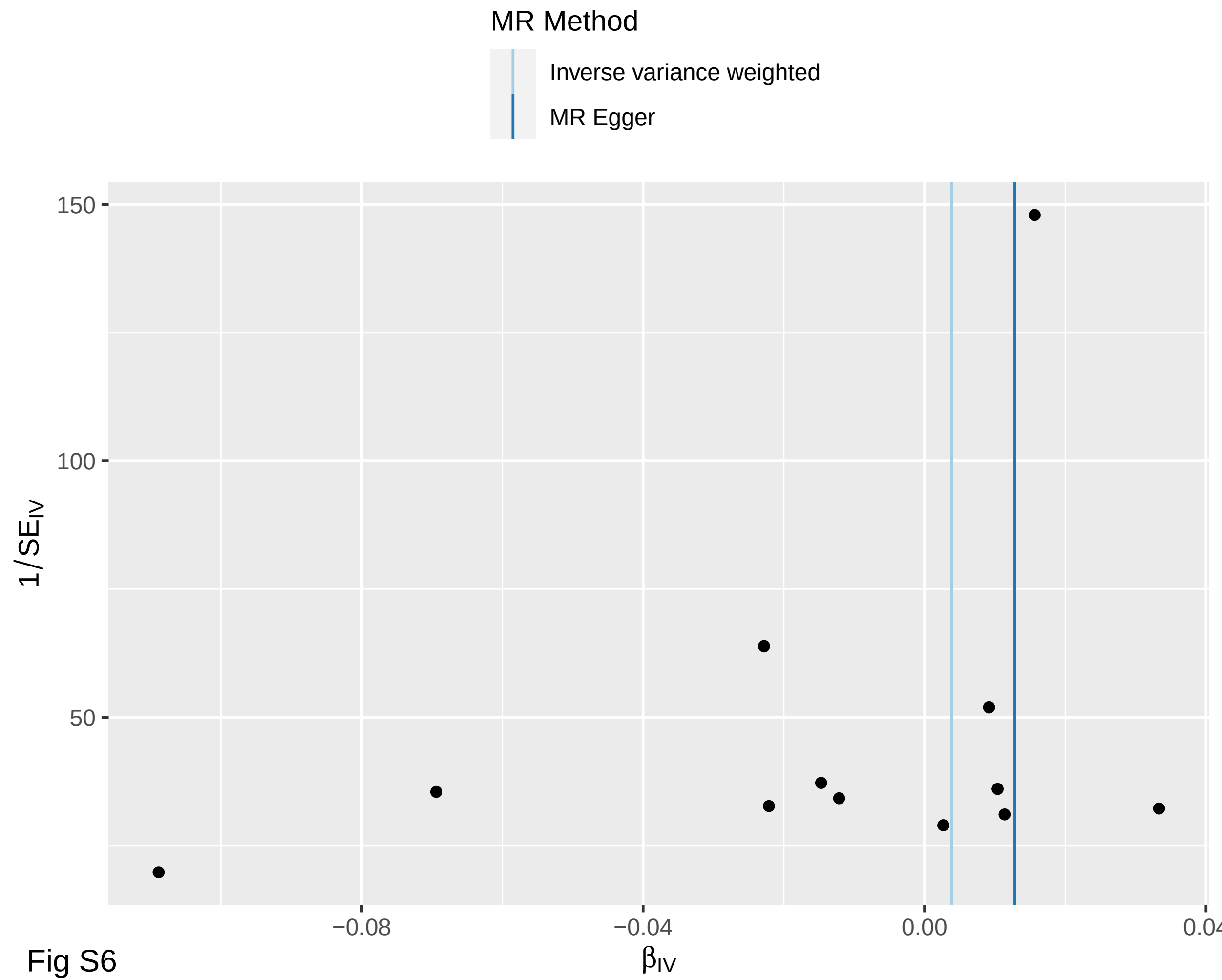
MR Method
Inverse variance weighted
MR Egger
150
100
50
1/SE$_{IV}$
−0.08
−0.04
0.00
0.04
β$_{IV}$

Fig S6

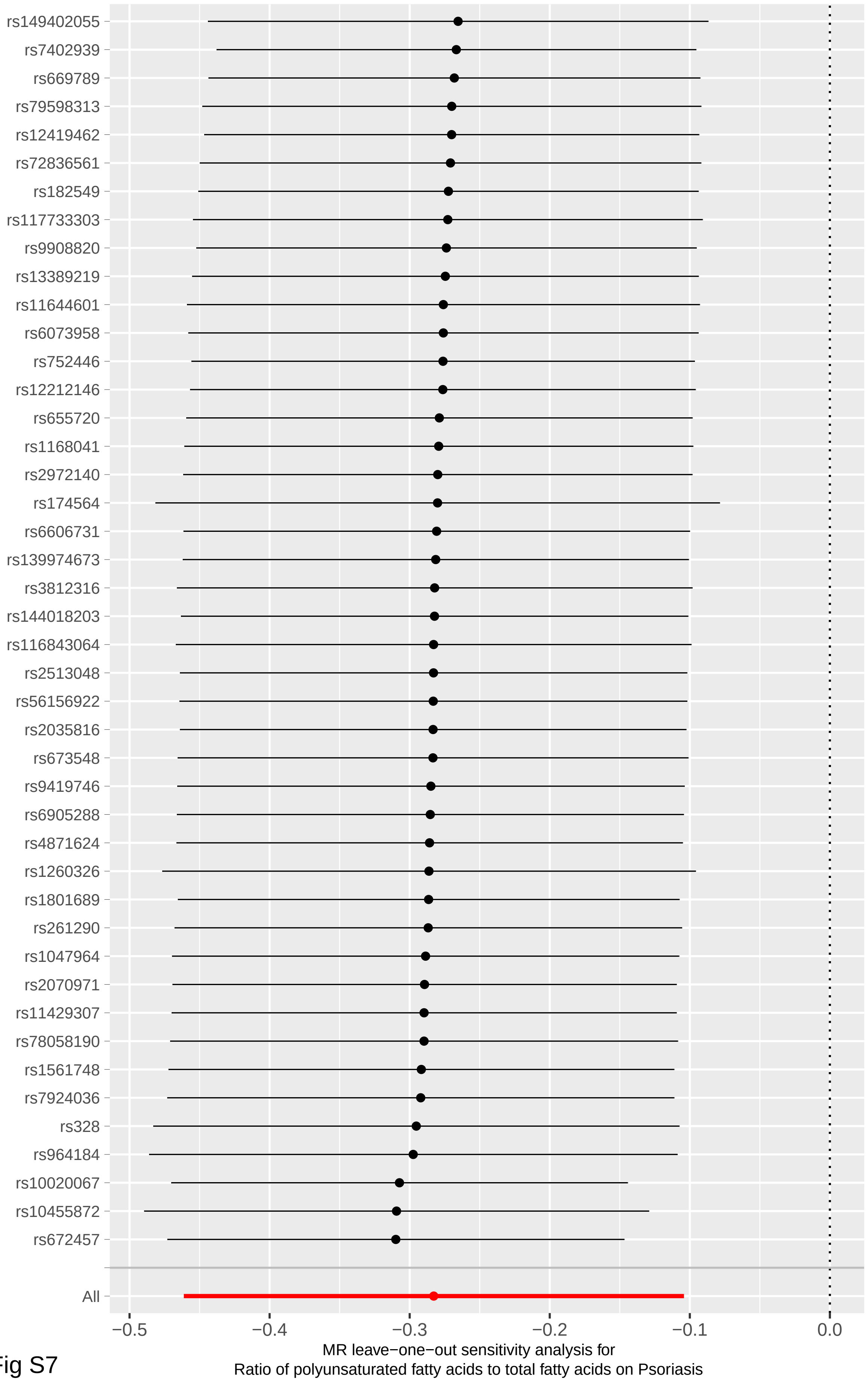
rs149402055
rs7402939
rs669789
rs79598313
rs12419462
rs72836561
rs182549
rs117733303
rs9908820
rs13389219
rs11644601
rs6073958
rs752446
rs12212146
rs655720
rs1168041
rs2972140
rs174564
rs6606731
rs139974673
rs3812316
rs144018203
rs116843064
rs2513048
rs56156922
rs2035816
rs673548
rs9419746
rs6905288
rs4871624
rs1260326
rs1801689
rs261290
rs1047964
rs2070971
rs11429307
rs78058190
rs1561748
rs7924036
rs328
rs964184
rs10020067
rs10455872
rs672457
All
−0.5
−0.4
−0.3
−0.2
−0.1
0.0
MR leave−one−out sensitivity analysis for
Ratio of polyunsaturated fatty acids to total fatty acids on Psoriasis

Fig S7

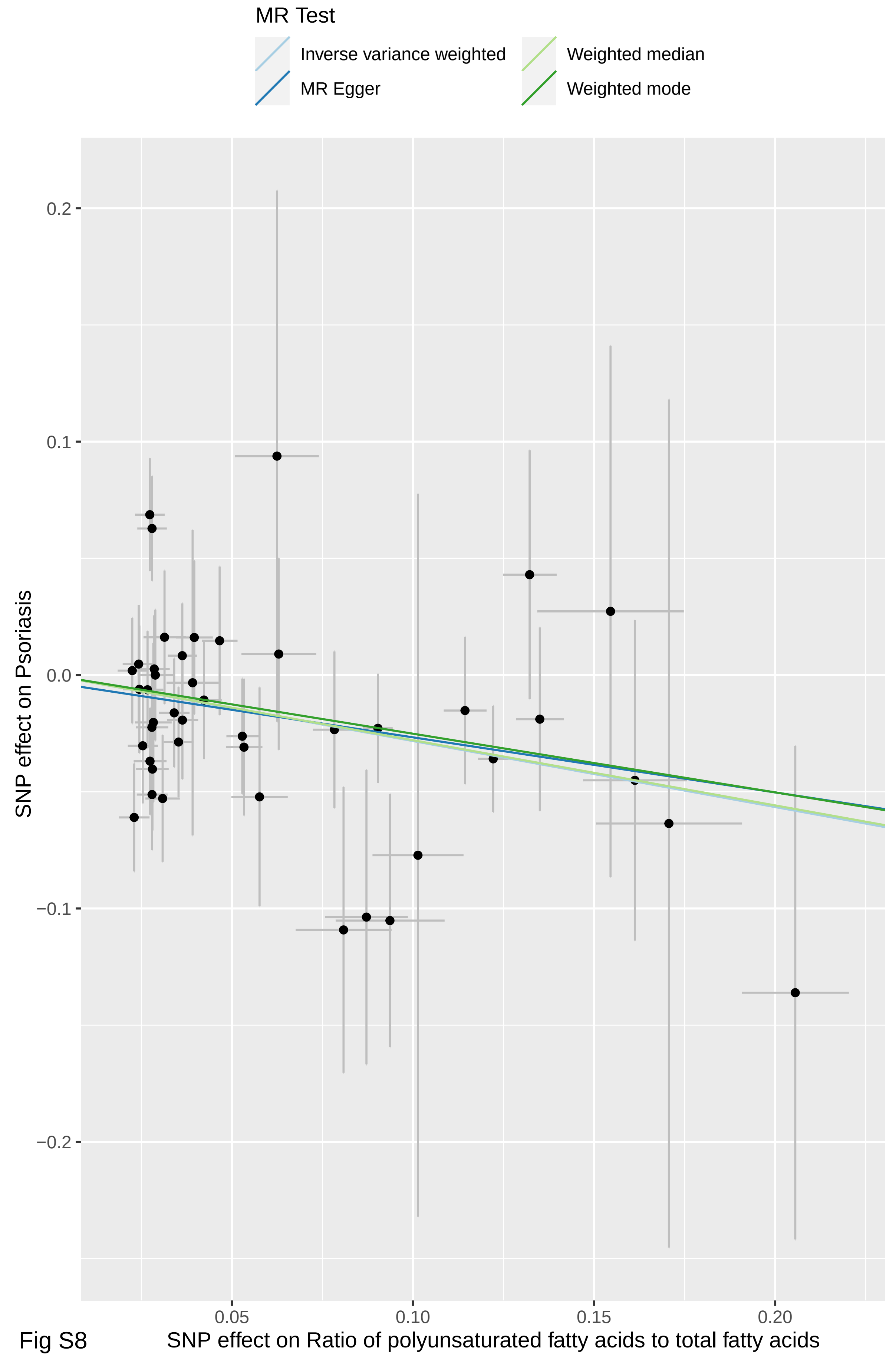
MR Test
Inverse variance weighted
MR Egger
Weighted median
Weighted mode
SNP effect on Psoriasis
0.2
0.1
0.0
−0.1
−0.2
0.05
0.10
0.15
0.20
SNP effect on Ratio of polyunsaturated fatty acids to total fatty acids

Fig S8

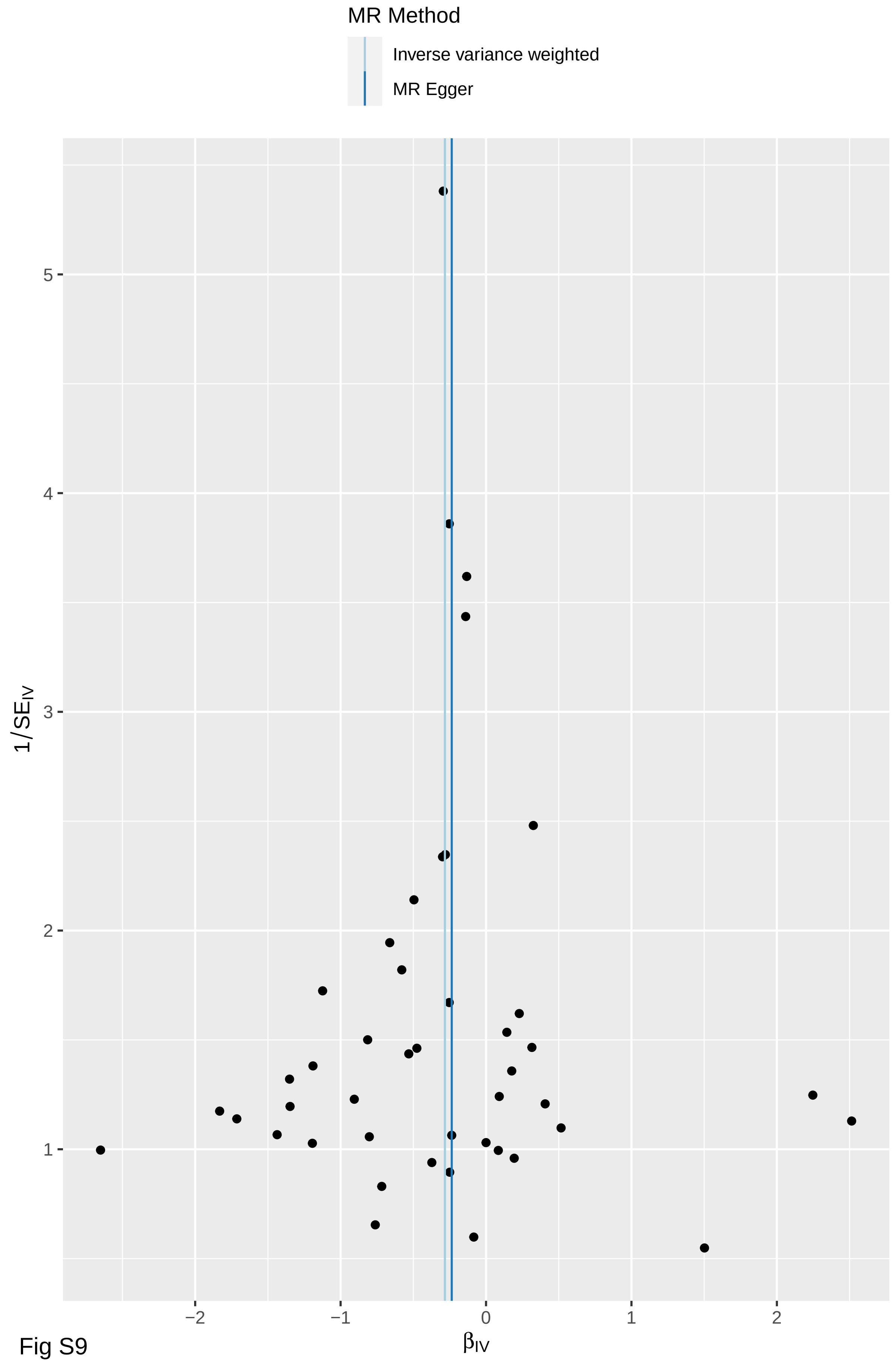

MR Method
Inverse variance weighted
MR Egger
5
4
3
2
1
1/SE$_{IV}$
−2
−1
0
1
2
β$_{IV}$

Fig S9

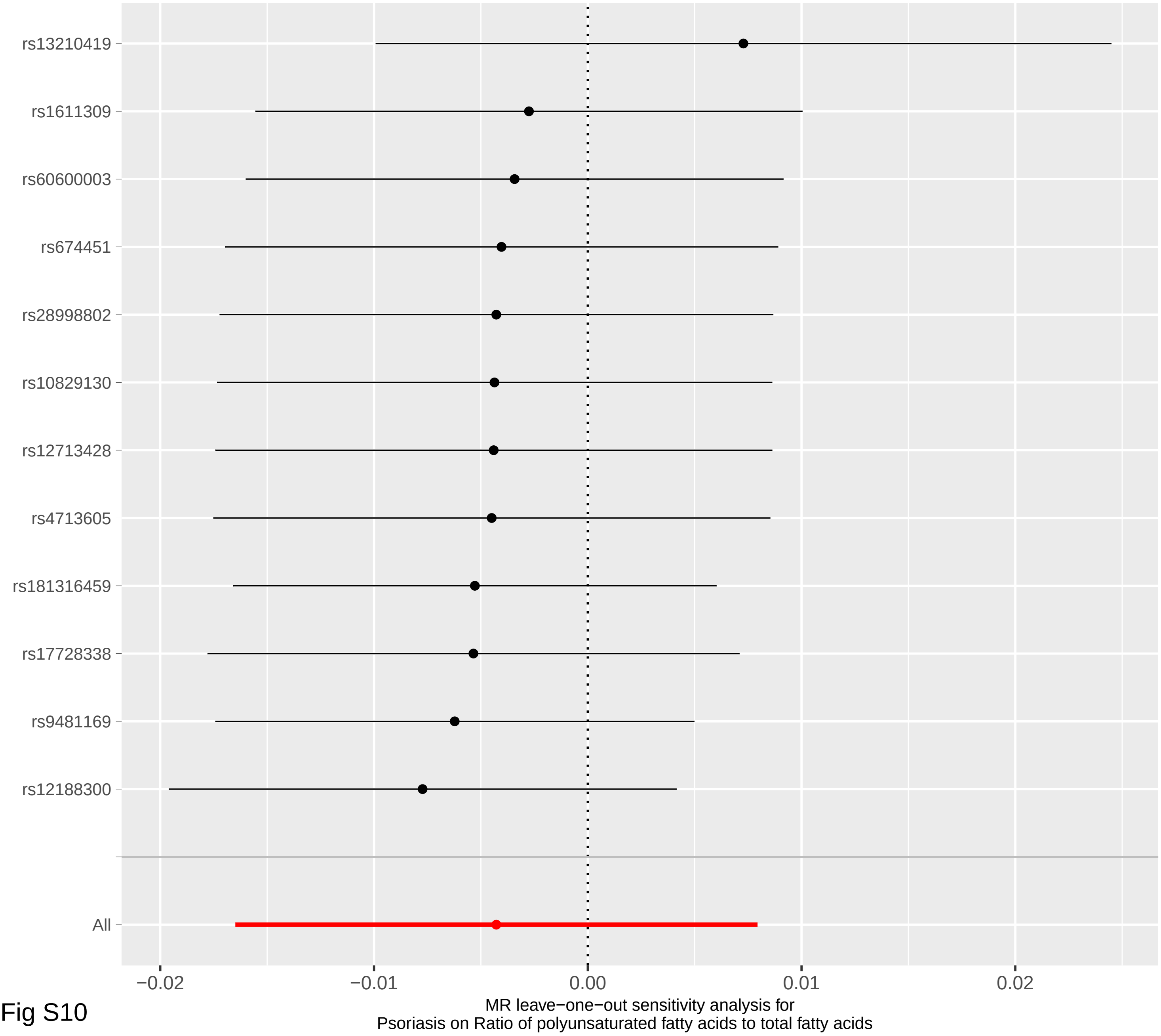

rs13210419
rs1611309
rs60600003
rs674451
rs28998802
rs10829130
rs12713428
rs4713605
rs181316459
rs17728338
rs9481169
rs12188300
All
−0.02
−0.01
0.00
0.01
0.02
MR leave−one−out sensitivity analysis for
Psoriasis on Ratio of polyunsaturated fatty acids to total fatty acids

Fig S10

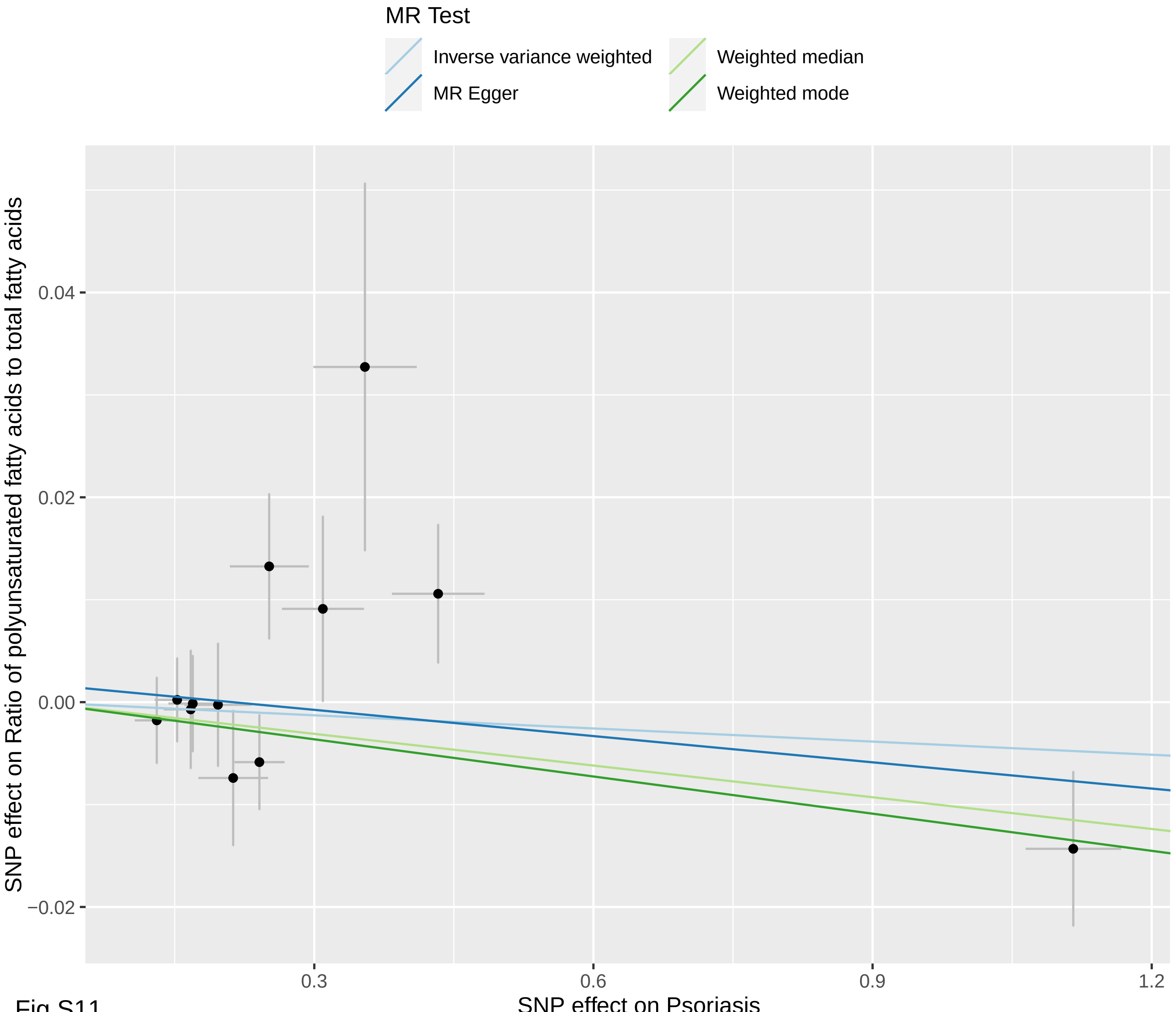

MR Test
Inverse variance weighted
MR Egger
Weighted median
Weighted mode
SNP effect on Ratio of polyunsaturated fatty acids to total fatty acids
0.04
0.02
0.00
−0.02
0.3
0.6
0.9
1.2
SNP effect on Psoriasis

Fig S11

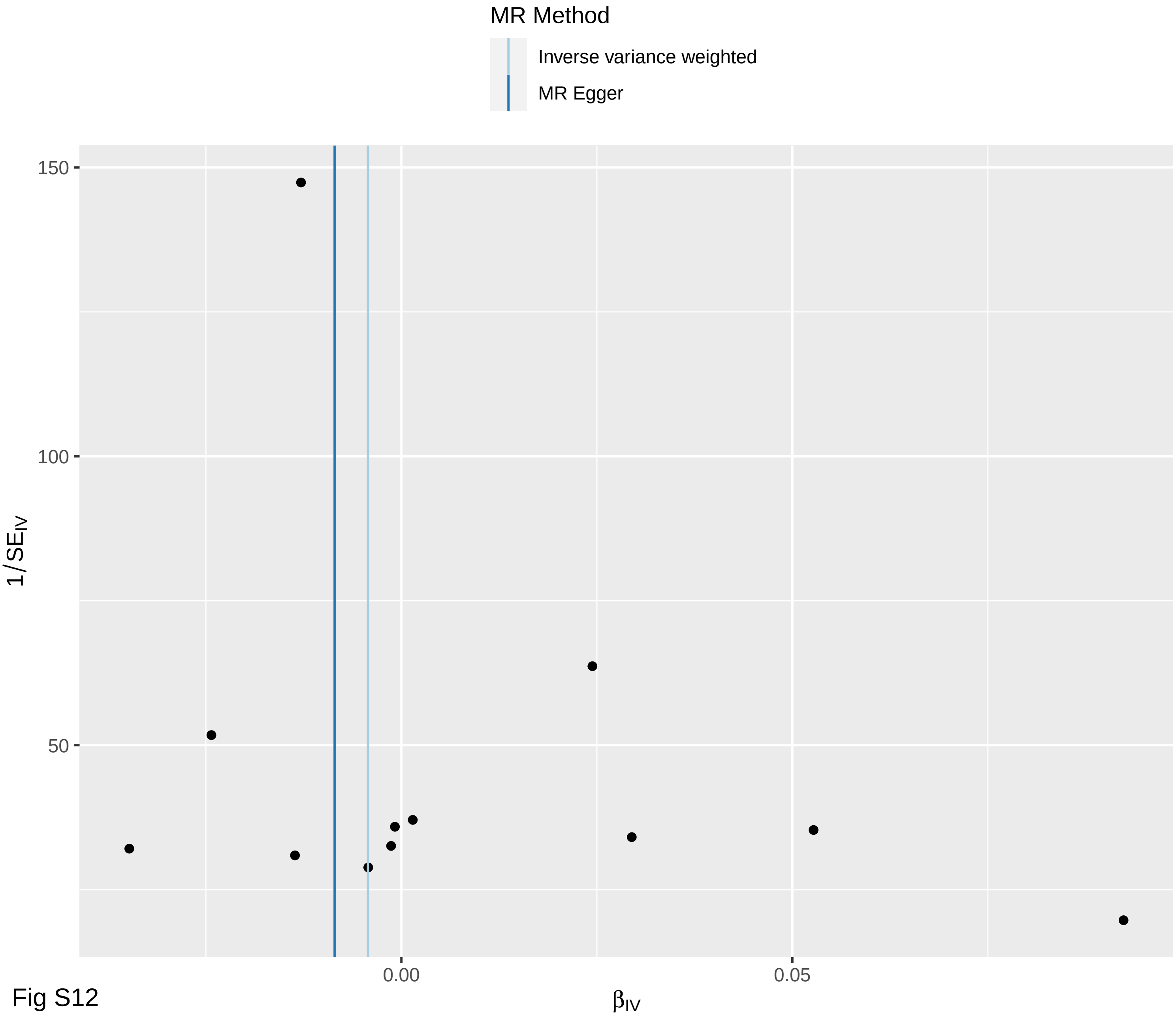
MR Method
Inverse variance weighted
MR Egger
150
100
50
1/SE_IV
0.00
0.05
β_IV

Fig S12

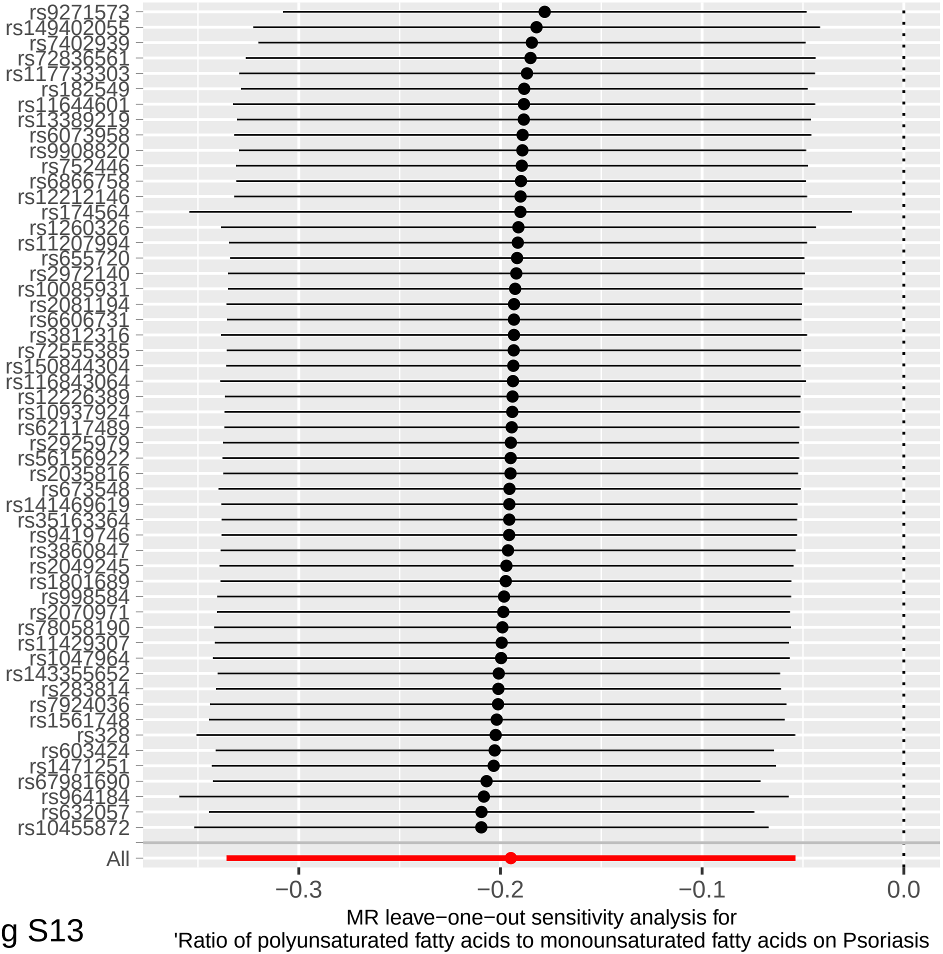
rs9271573
rs149402055
rs7402939
rs72836561
rs117733303
rs182549
rs11644601
rs13389219
rs6073958
rs9908820
rs752446
rs6866758
rs12212146
rs174564
rs1260326
rs11207994
rs655720
rs2972140
rs10085931
rs2081194
rs6606731
rs3812316
rs72555385
rs150844304
rs116843064
rs12226389
rs10937924
rs62117489
rs2925979
rs56156922
rs2035816
rs673548
rs141469619
rs35163364
rs9419746
rs3860847
rs2049245
rs1801689
rs998584
rs2070971
rs78058190
rs11429307
rs1047964
rs143355652
rs283814
rs7924036
rs1561748
rs328
rs603424
rs1471251
rs67981690
rs964184
rs632057
rs10455872
All
−0.3
−0.2
−0.1
0.0
MR leave−one−out sensitivity analysis for
'Ratio of polyunsaturated fatty acids to monounsaturated fatty acids on Psoriasis

Fig S13

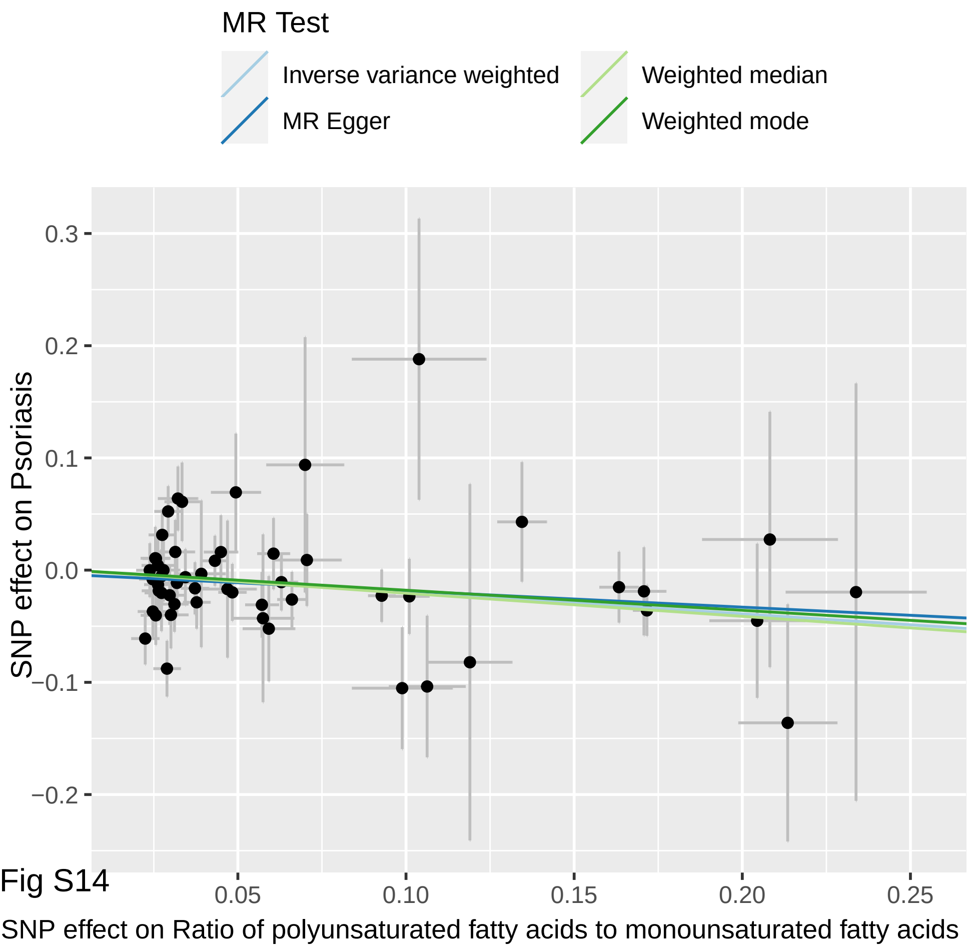

MR Test
Inverse variance weighted
MR Egger
Weighted median
Weighted mode
SNP effect on Psoriasis
0.3
0.2
0.1
0.0
−0.1
−0.2
0.05
0.10
0.15
0.20
0.25
SNP effect on Ratio of polyunsaturated fatty acids to monounsaturated fatty acids

Fig S14

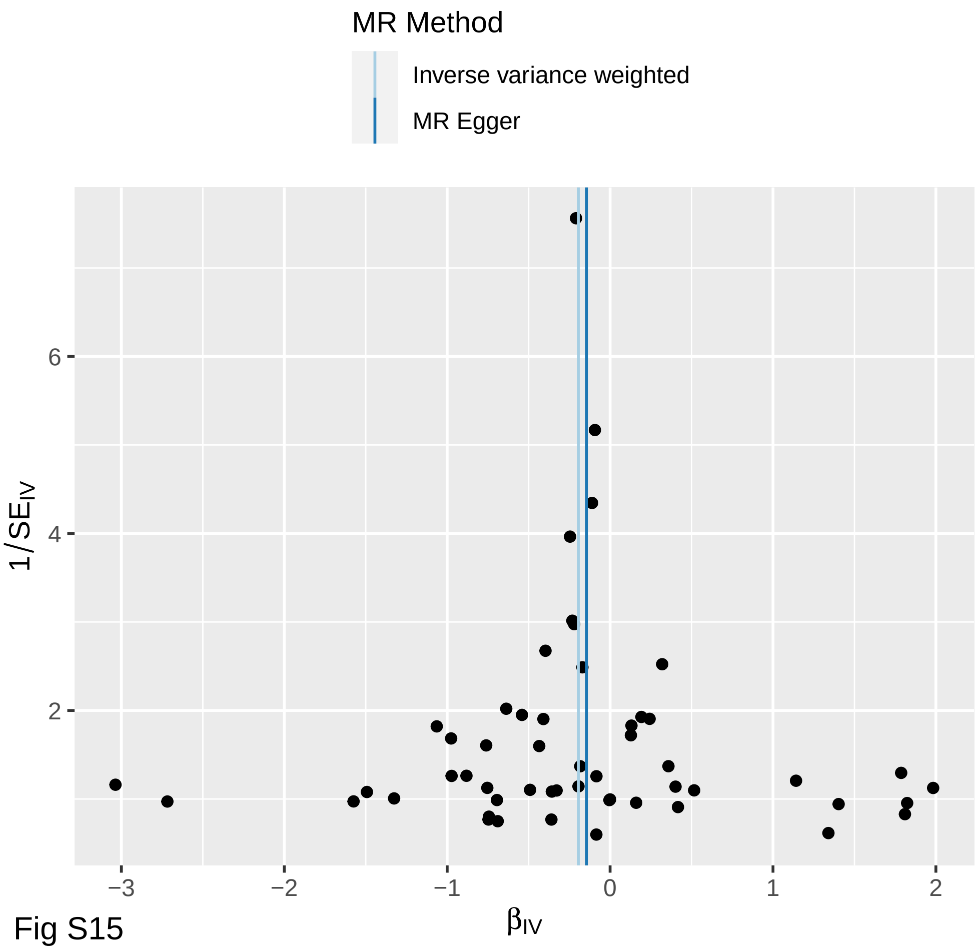
MR Method
Inverse variance weighted
MR Egger
6
4
2
1/SE_{IV}
−3
−2
−1
0
1
2
β_{IV}

Fig S15

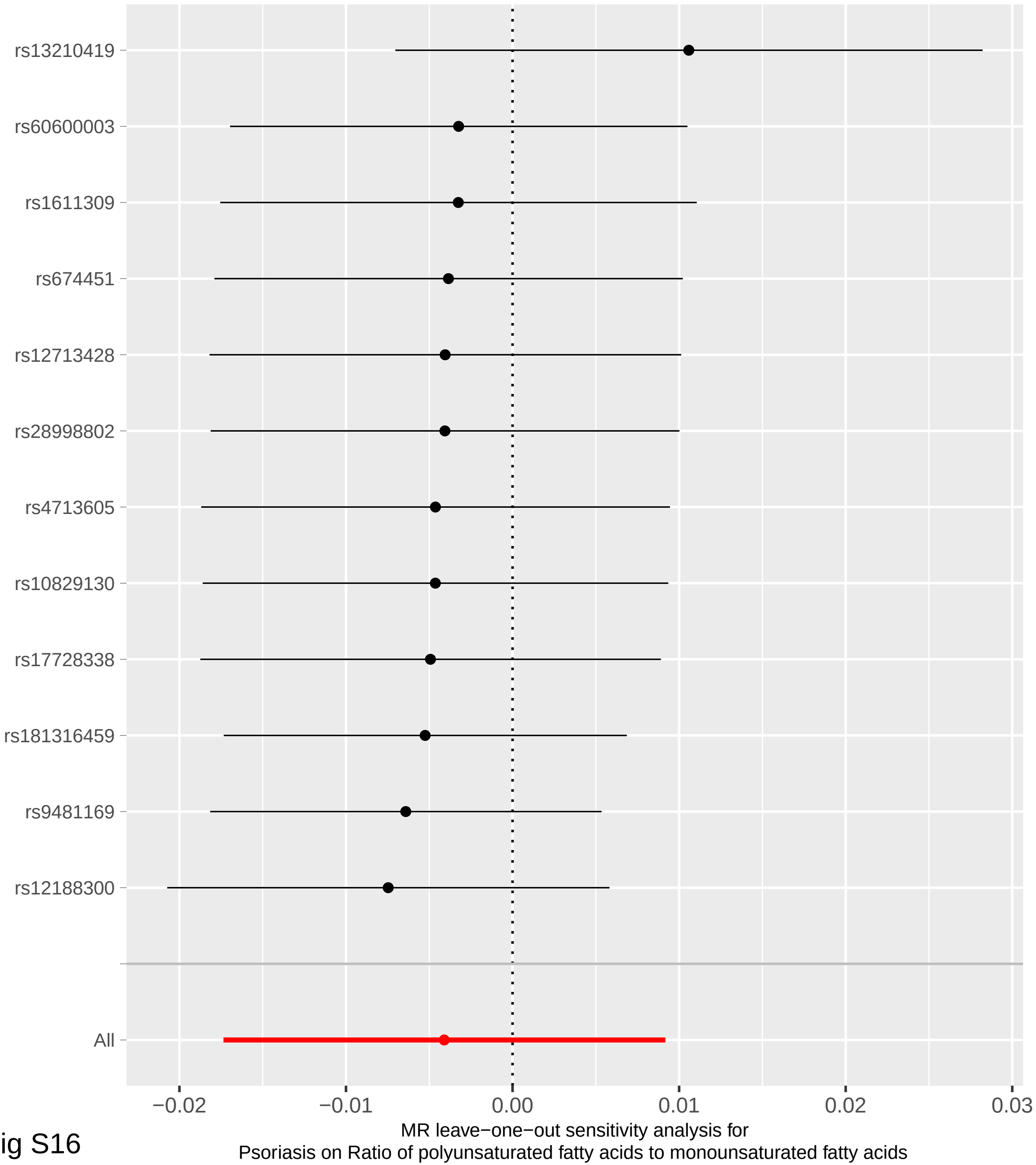
rs13210419
rs60600003
rs1611309
rs674451
rs12713428
rs28998802
rs4713605
rs10829130
rs17728338
rs181316459
rs9481169
rs12188300
All
−0.02
−0.01
0.00
0.01
0.02
0.03
MR leave−one−out sensitivity analysis for
Psoriasis on Ratio of polyunsaturated fatty acids to monounsaturated fatty acids

Fig S16

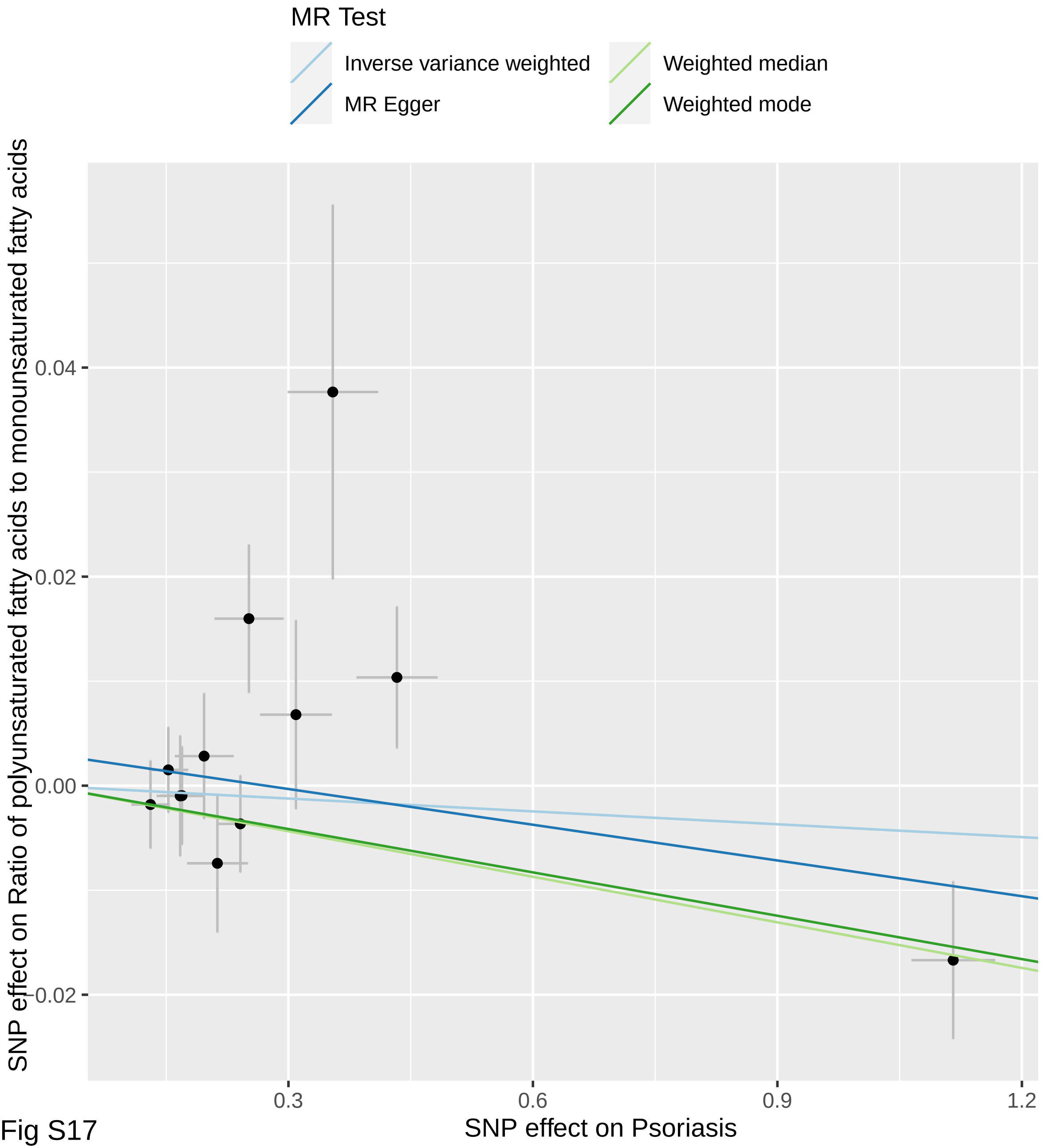
MR Test
Inverse variance weighted
Weighted median
MR Egger
Weighted mode
SNP effect on Ratio of polyunsaturated fatty acids to monounsaturated fatty acids
0.04
0.02
0.00
-0.02
0.3
0.6
0.9
1.2
SNP effect on Psoriasis

Fig S17

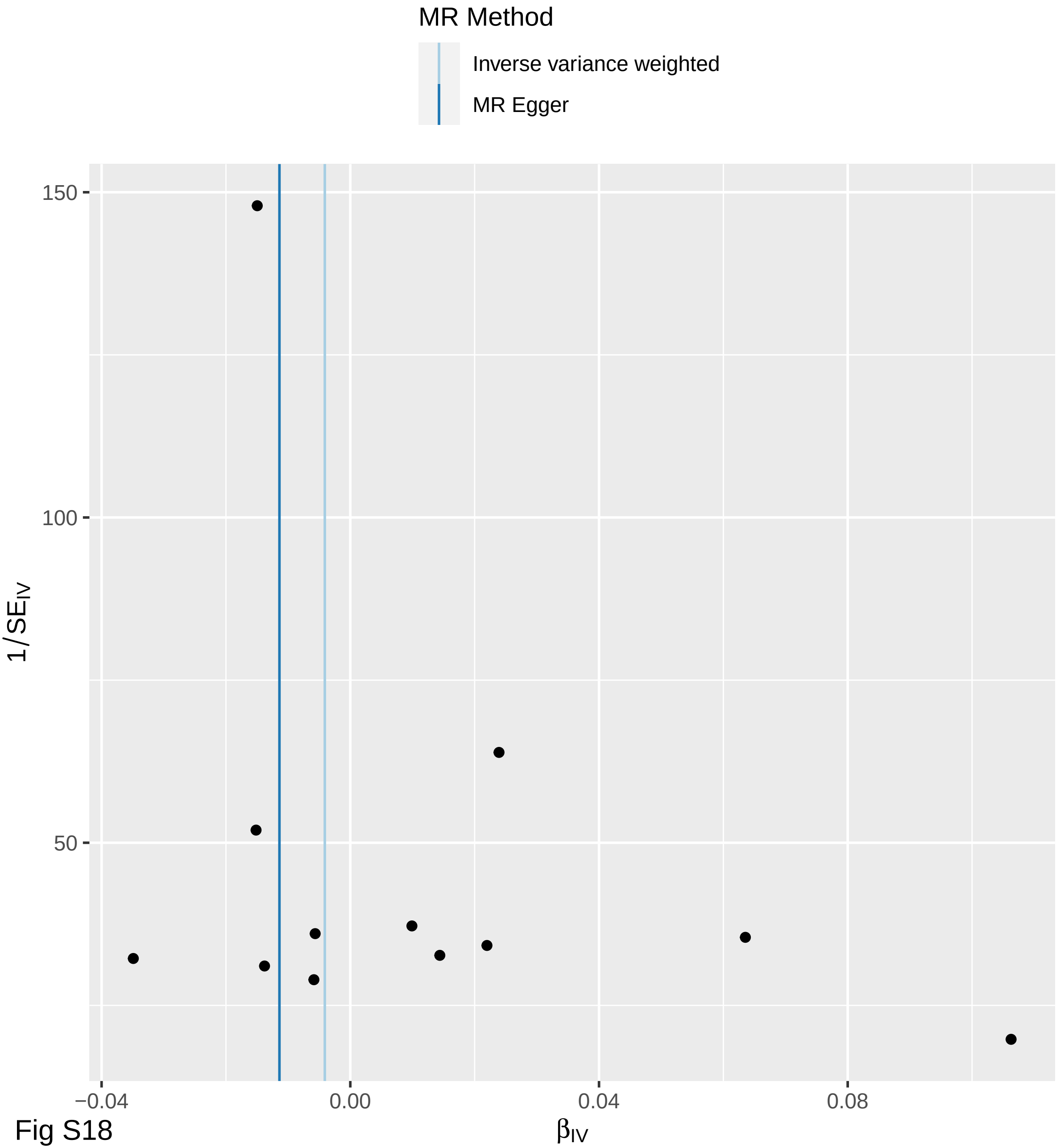
MR Method
Inverse variance weighted
MR Egger
150
100
50
1/SE_{IV}
−0.04
0.00
0.04
0.08
$β_{IV}$

Fig S18
